# ER stress inhibition enhances formation of triacylglcerols and protects endothelial cells from lipotoxicity

**DOI:** 10.1186/s12964-024-01682-y

**Published:** 2024-06-03

**Authors:** Igor Kovacevic, Paula Henriette Schmidt, Annkatrin Kowalski, Bernd J. Helms, Chris H. A. van de Lest, Alexander Kluttig, Guido Posern

**Affiliations:** 1 Institute of Physiological Chemistry, Medical Faculty, Martin Luther University Halle-Wittenberg, 06114 Halle (Saale), Germany; 2 Department Biomolecular Health Sciences, Veterinary Medicine, Utrecht University, Utrecht, 3584CM The Netherlands; 3 Institute of Medical Epidemiology, Biostatistics, and Informatics, Medical Faculty, Martin Luther University Halle-Wittenberg, Halle (Saale), Germany

**Keywords:** Endothelial dysfunction, ER stress, Lipidomic analysis, Ceramides, Obesity

## Abstract

Elevated concentrations of palmitate in serum of obese individuals can impair endothelial function, contributing to development of cardiovascular disease. Although several molecular mechanisms of palmitate-induced endothelial dysfunction have been proposed, there is no consensus on what signaling event is the initial trigger of detrimental palmitate effects. Here we report that inhibitors of ER stress or ceramid synthesis can rescue palmitate-induced autophagy impairment in macro- and microvascular endothelial cells. Furthermore, palmitate-induced cholesterol synthesis was reverted using these inhibitors. Similar to cell culture data, autophagy markers were increased in serum of obese individuals. Subsequent lipidomic analysis revealed that palmitate changed the composition of membrane phospholipids in endothelial cells and that these effects were not reverted upon application of above-mentioned inhibitors. However, ER stress inhibition in palmitate-treated cells enhanced the synthesis of trilglycerides and restored ceramide levels to control condition. Our results suggest that palmitate induces ER-stress presumably by shift in membrane architecture, leading to impaired synthesis of triglycerides and enhanced production of ceramides and cholesterol, which altogether enhances lipotoxicity of palmitate in endothelial cells.

**Supplementary Information:**

The online version contains supplementary material available at 10.1186/s12964-024-01682-y.

## Introduction

The World Health Organization (WHO) estimated that 650 million people in 2016 were obese. This medical condition is recognized as a major risk factor for cardiovascular disease (CVD), associated with a threefold increase in cardiovascular morbidity [1]. Impaired metabolism and excessive caloric intake in obesity leads to enhanced levels of circulating lipids, which can damage the endothelium and finally result in cardiac dysfunction, hypertension and ischemic heart disease, potentially with a fatal outcome. Endothelial dysfunction induced by excess levels of lipids in the circulation is strongly linked to increased oxidative stress and inflammatory signaling, common in metabolic syndrome, obesity and diabetes [2]. For instance, increased levels of triglycerides are negatively correlated with flow-induced dilation in humans [3]. Furthermore, ceramides are also increased in obese individuals [4] and C16:0, C18:0 and C24:1 ceramide species were associated with CVD in several patient cohorts [5]. Mechanistically, Luukonen at al. found that diet enriched with saturated fat elevated levels of serum ceramides [6]. Moreover, increased plasma levels of non-esterified fatty acids, also called free fatty acids (FFA), are characteristic for obesity and diabetes type 2.

FFAs are released from adipose tissue, but can also originate from dietary fat processing and inefficient uptake by adipose tissue [1]. Besides human and animal studies, in vitro experiments have demonstrated that palmitate (C16:0) and products from the lipolysis of triglyceride-rich lipoproteins can directly impair endothelial barrier integrity in microvascular endothelial cells and human aortic endothelial cells [7, 8]. According to studies, mostly performed in macrophages, the obesity-dependent inflammatory response in cells is initiated by long-chain saturated fatty acid-mediated activation of Toll-Like Receptors (TLRs) and subsequent enhanced expression of pro-inflammatory cytokines [9–11]. More specifically, experiments in mice have shown that FFAs activate TLR signaling in endothelial cells, thereby triggering an upregulation of leukocyte adhesion receptors, production of inflammatory cytokines and impaired glucose metabolism [12]. Alternatively, Lancaster et al. proposed that TLRs might not represent primary receptors for saturated fatty acids (SFAs) in macrophages [13]. In addition to TLR-mediated signaling, several alternative mechanisms of SFA signal transduction have been postulated such as the ER stress induction due to changes in protein palmitoylation or membrane composition [14, 15]. Nonetheless, there is still no consensus on what signaling event is the initial trigger for the SFA-induced activation and lipotoxicity, i.e. the abberrant lipid accumulation and cytopathic effect in endothelial cells.

The aim of this study was to compare signaling pathways and morphological changes induced by selected FFAs in macro- and microvascular endothelial cells with focus on on lipid droplet formation, endoplasmic reticulum (ER) stress signaling and changes in lipid metabolism. In contrast to oleic acid (cis C18:1 (n-9)) which enhanced formation of lipid droplets, C16:0 (palmitic acid) induced an ER stress dependent unfolded protein response (UPR) and impaired autophagy. The ER stress inhibitor 4-PBA (4-phenylbutyrate) reverted the effects of palmitate on both processes, partially through enhancement of lipid droplet formation in palmitate-treated cells. Additionally, cholesterol synthesis was also enhanced by palmitate and this effect was as well abrogated upon 4-PBA co-treatment. Small-scale siRNA screening suggested that ceramide synthesis might be crucial for the effects of palmitate on ER stress induction and autophagy blockage in endothelial cells. In accordance with these results, inhibition of ceramide synthesis by application of serine palmitoyl transferase inhibitor myriocin, rescued all above-mentioned effects of palmitate in endothelial cells. Using lipidomic analysis we found that palmitate treatment of endothelial cells promoted synthesis of phosphatidic acid and general saturation of acyl chains within phospholipids and incorporation of palmitate into these. Lipidomic analysis also confirmed our initial observation that 4-PBA enhances the synthesis of triglycerides in palmitate-treated cells. Our study contributes to decipher the SFA-induced changes in lipid metabolism and subsequently activated signaling pathways in endothelial cells.

## Materials and methods

### Inhibitors

Myriocin (#63,150) and A922500 (#10,012,708) were purchased from Caymann Chemicals, PF-06424439 (#PZ0233) and 4-PBA (#SML0309) from Sigma, and Propranolol (#BML-ST405-0005) was obtained from Enzo Life Sciences.

### Antibodies

Following antibodies were used in this study: mouse GAPDH (#G8795, Sigma), rabbit LC3B (#3868, Cell Signaling Technology), rabbit RhoB (#63,876, Cell Signaling Technolgy), rabbit p62 (#5114, Cell Signaling Technology), mouse p62 (#sc-48,402, Santa Cruz), mouse Vinculin (#V9131, Sigma), mouse CHOP (#2895, Cell Signaling), rabbit p44/42 MAPK (Erk1/2)(#4695, Cell signaling), rabbit pp38 (#9211, Cell Signaling), rabbit Grp78 (#SAB4501452, Sigma), and rabbit Perilipin 2/ADRP (#15294-1-AP, Proteintech). Following secondary antibodies were used in this study: HRP-linked anti-mouse (#7076, Cell Signaling) and anti-rabbit antibody (#7074, Cell Signaling), anti-mouse Alexa 488 (#A-11,094, Invitrogen), anti-rabbit Alexa 647 (#A-21,244, Invitrogen) and anti-mouse Alexa 546 antibody (#A-10,040, Invitrogen).

### Cell culture

#### HUVEC

Primary human umbilical vein endothelial cells (HUVEC) were purchased from Lonza (#CC-2519) and cultured in Endothelial Cell medium (ECM) from ScienCell (#1001) supplemented with 5% FCS (Fetal Calf Serum), Endothelial Cell Growth Supplement and penicilin/streptomycin antibiotics. Cells were cultured at 37 °C and 5% CO2 and the medium was refreshed every second day. Experiments were performed with HUVEC until passage 5.

#### HMVEC

Human Lung Microvascular Endothelial Cells (HMVEC) were obtained from Sciencell Research Laboratories (#sc-3000) and cultured in Endothelial Cell Medium (ECM)(#sc-1001). Cells were cultured at 37 °C and 5% CO2 and the medium was refreshed every second day. Experiments were performed with HMVEC until passage 3.

#### THP-1

THP-1 human monocytic leukemia cell line was a generous gift from Rüdiger Horstkorte´s laboratory (Institute of Physiological Chemistry, MLU Halle (Saale)). THP-1 cells were cultured in RPMI (GIBCO) supplemented with 2mM L-Glutamine (GIBCO), Non-essential amino acids (GIBCO) and 10% FCS. To maintain the THP-1 cells in cullture, the cell suspension was diluted with the fresh cell culture medium every second day.

### Western blot

For western blot experiments 150.000 endothelial cells were seeded per well of the 12-well cell culture plate 24 h prior to stimulation with fatty acids. After stimulation and prior to lysis cells were washed PBS supplemented with 1mM CaCl2 and 0.5 mM MgCl2 and then lysed in 2x SDS sample buffer (SB) (125 mM Tris-HCl, 2% SDS, 20% glycerol, 100 mM DTT, 0.02% bromophenol blue in H_2_O). Equal volumes of lysates were loaded on polyacrylamide gel and proteins were separated by SDS-PAGE and transferred to nitrocellulose membrane, followed by incubation with designated primary antibodies in 5% bovine serum albumin (BSA) in TBS-T (Tris-buffered saline with 0.1% Tween). Protein bands were visualized with enhanced chemiluminescence reagent (Amersham/GE-healthcare) on the ChemiDOC MP Imaging System (BioRad). Quantification of western blots was performed using ImageJ.

### Preparation of fatty acid stock solutions

Sodium palmitate (#P9767), sodium oleate (#O7501), sodium laurate (#L9755), docosahexaenoic acid sodium salt (#D8768), sodium myristate (#M8005), elaidic acid (#E4637) and sodium stearate (#S3381) were all purchased from Sigma. Fatty acids solution was prepared as described previously [16]. In brief, fatty acids were dissolved in sterile water by heating to 70 °C and vortexing to prepare 100 mM stock solutions. 100 mM stock solutions were immediately diluted to 5mM concentration in sterile filtered 5% w/v BSA/DMEM solution (Fatty acid free BSA (#A-6003, Sigma)) pre-warmed at 40° C yielding stock solution with fatty acid: BSA molar ratio of 6.6:1. Before stimulation, 5mM stock solution was incubated for 1 h at 40° C with mixing at 800 rpm prior to adding to the endothelial cell medium at final concentration of 150 µM. 5% BSA/DMEM solution was used as vehicle control.

### Immunofluorescence staining

HUVECs or MVEC were seeded on fibronectin-coated (5 µg/ml) 2 cm2 coverslips (Thermo Scientific, Menzel-gläser) (#10,319,303). Warm (37 °C) 4% paraformaldehyde (Sigma Aldrich) (#158,127) in phosphate buffered saline (PBS) was added onto the cells and incubated at room temperature for 15 min. After three washes with PBS, cells were permeabilized with 0.2% Triton X-100 in PBS for 3 min and blocked for 30 min with 1% BSA in PBS. Upon blocking step coverslips were stained with primary antibodies in 1% BSA/PBS for 1 h at room temperature. After 3X washing with PBS, coverslips were incubated with fluorescently-labelled secondary antibodies (1:200 in 1% BSA/PBS), Alexa 647-conjugated phalloidin (Invitrogen) and 4′,6-diamidino-2-phenylindole (DAPI) (Thermo Fisher Scientific) for 1 h at room temperature. Finally, coverslips were were washed again in PBS and mounted on Mowiol/ 1,4-diazabicyclo[2.2.2]octane (DABCO) solution. Fluorescent microscopy images were obtained using Zeiss AxioObserver Z1 microscope equipped with structured illumination ApoTome.2 module. Images were analyzed and equally adjusted with ImageJ software.

### siRNA transfection

One day prior to transfection HUVECs were seeded on fibronectin coated glass coverslips on 12-well cell culture plates or 96-well plates with transparent bottom. On the day of transfection cells were 70–80% confluent. Transfection was performed using Dharmafect I reagent (Dharmacon/Horizon Discovery) and 20 nM of one of the ON-TARGET siRNA-SMART pools (Dharmacon/Horizon Discovery) targeting following genes: PTDSS2, PTDSS1, EPT1, CHPT1, SGMS1, SGMS2, CDIPT, CRLS1, LPIN1, LPIN2, LPIN3, CDS1, CDS2, DGAT1, DGAT2, SOAT1, SOAT2, FDFT1, HMGCR, CERS1, CERS2, CERS3, CERS4, CERS5, CERS6, B4GALT6, B4GALT5, SQSTM1 (p62) or DDIT3 (CHOP). ON-TARGET plus Non-targeting Control pool was used as a negative control. Transfected cells were used for experiments at 48 h post-transfection.

### Triglyceride and cholesterol quantification in endothelial cells

Triglycerides were quantified using Triglyceride quantification kit obtained from Sigma (#MAK266) according to manufacturer’s instructions. In brief, 500.000 HUVEC were seeded per well of the 6-well plate and 24 h later the cells were treated with fatty acids in combination with inhibitors for 16 h. Triglycerides were extracted from treated cells in 5% NP-40 solution and measured fluorometrically at λ_ex_535/ λ_ex_587 nm using CLARIOStar plate reader.

Cholesterol was measured using Cholesterol/Cholesteryl Ester Assay Kit from Abcam (#ab65359). The HUVEC were seeded and treated as described above for triglyceride quantification. Upon treatment the lipids were extracted using Chloroform: Isopropanol: NP-40 (7:11:0.1) extraction buffer according to manufacturer’s instructions and cholesterol content was determined by measuring of fluorescence intensity of the cholesterol probe at λ_ex_535/ λ_ex_587 nm using CLARIOStar microplate reader.

### Fillipin III staining

Fillipin III was purchased from Sigma (#SAE0087) and used for staining and quantification of free cholesterol content in HUVEC and MVEC. For this assay 40.000 HUVEC or MVEC were seeded on fibronectin coated 8-well Ibidi slides (#80,826). Next day the cells were treated with fatty acids in combination with inhibitors for additional 16 h. After stimulation the cells were fixed with 4% PFA in PBS supplemented with 1mM CaCl2 and 0.5 mM MgCl2 for 10 min, washed with PBS and permeabilized with permeabilization buffer (0.05% Triton X-100, 3% BSA in PBS). Upon permeabilization the cells were incubated in 25 µg/ml Fillipin III solution in dark for 30 min, washed with PBS and immediately imaged using Zeiss AxioObserver Z1 microscope.

### ELISA analysis of human serum samples from CARLA Study

Serum samples for enzyme-linked immunoSorbent assay (ELISA) analysis were obtained from CARdiovascular disease, Living and Ageing in Halle (CARLA) Study [17] at the department of Medical Epidemiology, Biostatistics, and Informatics (IMEBI), Martin-Luther-University, Halle-Wittenberg. The participant’s data and samples were collected at the baseline in the period 2002–2006, with the first follow-up in 2007–2010 and second follow-up in 2013. Data collected within the CARLA study include anthropometric information (e.g. body mass index (BMI), waist circumference, etc.), as well as serum lipid profiles and other parameters. Permission to frozen serum samples from CARLA study and access participants data in anonymous manner has been approved by the steering committee of the CARLA study and the ethical committee of the Medical Faculty at the Martin-Luther-University Halle-Wittenberg (Reference number: 2019-12).

Sera of 20 control individuals (BMI 18.5-24.99) and 20 obese individuals (BMI 30.0-34.99) were selected for the ELISA-based quantification of CHOP, Grp78 and p62 proteins. Metabolic parameters of selected individuals are listed in the Supplementary Table 1. Participants with diabetes were excluded from this analysis as well as individuals treated with lipid-lowering drugs, cancer patients or individuals with alcohol consumption over 25 g/day. There were no significant differences between groups in serum triglycerides, total cholesterol or low-density lipoprotein (LDL) concentration, although cholesterol/high-density lipoprotein (HDL) ratio was increased in obese group when compared to healthy weight group due to decreased HDL concentration in the obese group. Furthermore, selected participants had equal sex and age distribution and similar smoking habits. Serum samples underwent equal freezing and thawing cycles.

Following ELISA kits were used for serum analysis: Grp78/BiP ELISA kit from ENZO (# ADI-900-214-0001), P62/SQSTM1 BioAssay(TM) ELISA Kit from Biomol (# 519410.96) and Human DNA Damage Inducible Transcript 3 (CHOP) ELISA Kit from Biozol (#MBS733941). All assays were performed in duplicates according to manufacturer’s instructions. Serum samples were diluted 1:5 for p62/SQSTM1 quantification and 1:20 for Grp78/BiP quantification.

### Transfection of HUVEC with the ER-Scarlet marker

For the morphological analysis of the ER structure HUVEC were seeded on fibronectin-coated glas coverslips and 24 h later at 70% confluency the cells were transfected with the ER-mScarletI plasmid. ER-mScarletI was a gift from Dorus Gadella (Addgene plasmid #137,805; http://n2t.net/ addgene: 137,805; RRID: Addgene_137805). Transfection was performed using TransIT LT1 transfection reagent obtained from Mirus (#Mir2300). At 24 h post transfection, the cells were washed with PBS and fixed using warm (37 °C) 4% paraformaldehyde (Sigma Aldrich) (#158,127) in phosphate buffered saline (PBS). Microscopy analysis was performed using using the Zeiss AxioObserver Z1 microscope equipped with structured illumination ApoTome.2 module. Images were analyzed and equally adjusted with ImageJ software.

### Dextran leakage assay

Dextran leakage assay was performed using the 6-well Transwell inserts with 3 μm pores (Corning). Prior to cell seeding the Tranwells were coated with 5 µg/ml fibronectin (Sigma) in PBS solution for 1 h. Afterwards, 140.000 HUVEC were seeded per insert and incubated for additional 72 h. The cells were stimulated with 150 µM fatty acid solution in combination with inhibitors or with vehicle (BSA) alone for additional 16 h. Upon stimulation the cells were washed with 4-(2-hydroxyethyl)-1-piperazineethanesulfonic acid (HEPES) medium (HEPES 20 mM, NaCl 132 mM, KCl 6 mM, CaCl_2_ 1 mM, MgSO_4_ 1 mM, KH_2_PO_4_ 1.2 mM, Glucose 5 mM and 0.4% BSA) and 0.25 mg/ml of the 70 kDa fluorescein isothiocyanate (FITC)-Dextran (‘, 90718 Sigma) solution in HEPES medium was added to the upper chamber and empty HEPES medium to the lower chamber. After 30 min 3x technical replicates of 100 ul were collected the from lower chamber of each well and the fluorescence intensity was measured at 488nm wavelength using CLARIOStar plate reader.

#### THP-1 adhesion assay

For the adhesion assay 150.000 HUVEC were seeded per well of the 12-well cell culture plate. After 24 h the HUVEC were treated with BSA, palmitate (150 µM) or TNF-α (10 ng/ml) (Peprotech) for 16 h. Prior to adhesion assay 200.000 THP-1 cells per condition were labelled with 5 µM Calcein Red AM (Cayman Chemical (#20,632)) and washed twice in HEPES medium (HEPES 20 mM, NaCl 132 mM, KCl 6 mM, CaCl_2_ 1 mM, MgSO_4_ 1 mM, KH_2_PO_4_ 1.2 mM, Glucose 5 mM and 0.4% BSA). Labelled THP-1 cells were added to HUVECs and allowed to adhere for 30 min. Non-adherent THP-1 cells were removed and HUVEC were washed 5x times with pre-warmed HEPES medium. Afterwards the adherent THP-1 cells on HUVEC monolayers were imaged using EVOS FL Microscope at 546 nm emission wavelength. For quantification, Calcein Red AM- labelled THP-1 cells were counted per field of view.

### Lipid extraction

The extraction of lipids was carried out using a modified version of the Bligh & Dyer method [18]. Briefly, 800 µL of sample was placed in a glass tube and 2 mL of methanol and 1 mL of chloroform were added and mixed thoroughly. The mixture was incubated for 20 min followed by the addition of 2 mL of chloroform and 2 mL of deionized water. The resulting hydrophilic and hydrophobic phases were separated by centrifugation-during 5 min at 2000 ×g at room temperature. The hydrophobic bottom phase was carefully transferred to a new conical glass tube. To ensure complete lipid collection, the extraction of the remaining sample (hydrophilic phase) was repeated with an additional 2 mL of chloroform. Subsequently, the samples were dried by injection of nitrogen gas and stored under a nitrogen atmosphere at -20 °C.

### Phospholipids and lipid extraction

Dried lipid extracts were dissolved in 100 µL chloroform/methanol (1:1, v/v) and injected (10 µL) into a hydrophilic interaction liquid chromatography column (2.6 μm HILIC 100 Å, 50 × 4.6 mm, Phenomenex, CA). Lipid classes were separated by gradient elution on an Infinity II 1290 UPLC (Agilent, CA) at a flow rate of 1 ml/min. A mixture of acetonitrile and acetone (9:1, v/v) was used as solvent A, while solvent B consisted of a mixture of acetonitrile, H_2_O (7:3, v/v) with 50 mM ammonium formate. Both solvents A and B contained 0.1% formic acid (v/v). Gradient elution was performed as follows (time in min, %B): (0, 0), (1, 50), (3, 50), (3.1, 100), (4, 100). Between successive samples, the column was not re-equilibrated. The column outflow was connected to a heated electrospray ionisation source (hESI) of an Orbitrap Fusion mass spectrometer (Thermo Scientific, MA) operating at -3600 V in negative ionisation mode. The evaporator and ion transfer tube were set at 275 °C and 380 °C, respectively. Full scan measurements (MS1) in the mass range of 450 to 1150 amu were collected with a resolution of 120,000. Parallel data-dependent MS2 experiments were performed with HCD fragmentation set at 30 V, using the two-stage linear ion trap to generate up to 30 spectra per second.

### Neutral lipids

The dried lipids extracts were dissolved in 100 µL chloroform/methanol (1/1; v/v). The lipids (10 µL) were separated on a Halo C18 fused core column (3.0 × 150 mm, 2.7 μm; Advanced Materials Tech, Wilmington, DE) with a gradient from 100% methanol/water (1/1; v/v) to 100% methanol/2-propanol (8/2; v/v) in 2 min and an additional 5.5 min elution with methanol/2-propanol. The elution took place at 40 °C and a flow rate of 600 µL/min. The column outflow was connected to an atmospheric pressure chemical ionisation (APCI) source coupled to a Q Exactive HF mass spectrometer. Full scan measurements (MS1) in the mass range of 200 to 1100 amu were collected with a resolution of 120,000.

Data processing using R-script was based on the ‘XCMS’ package for peak recognition and integration [19]. Lipid classes were identified based on retention time and molecular species were then compared with a lipid database generated *in silico*, with a mass accuracy of less than 0.003 Da.

## Results

### Long-chain saturated free fatty acids effects on autophagy and ER stress in endothelial cells are restored by pharmacological ER stress inhibition

**Fig. 1:**
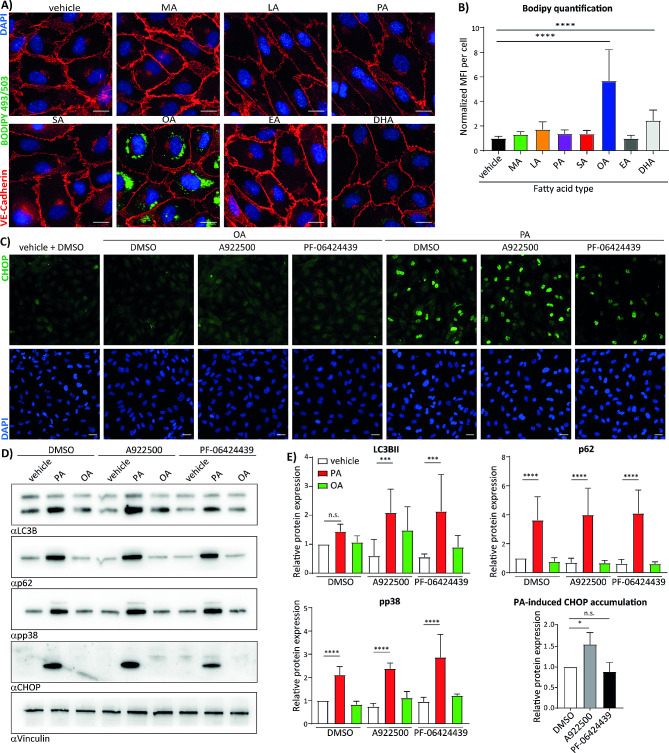
Saturated fatty acids induce impairment of autophagy and induction of UPR in endothelial cells. (**a**) BODIPY 493/503 staining of lipid droplets. HUVEC were seeded on 8-well IBIDI chamber and treated for 16 h with 150 µM of FFAs coupled to 5% BSA or BSA alone as a control. The cells were fixed, stained with BODIPY 493/503, anti-VE-Cadherin antibody and DAPI. The samples were imaged using Zeiss AxioObserver. Scale bar represents 15 μm. **(b)** Quantification of BODIPY staining from a). *n* = 3. **(c)** Immunofluorescence analysis of CHOP expression in endothelial cells. HUVEC were seeded on glass coverslips and treated for 16 h with 150 µM palmitate (PA) or oleate (OA) in combination with 5 µM A922500 or 10 µM PF-06424439. Treated cells were fixed and stained with anti CHOP antibody and DAPI. Scale bar represents 25 μm. **(d)** Immunoblot analysis of HUVEC treated as in c). Cells were lysed in 2x sample buffer and analysed for expression of LC3B, p62, pp38 and CHOP. Vinculin was used as loading control. **(e)** Quantification of immunoblots from d). All samples were normalized to control condition. *n* = 3. GraphPad Prism software and one-way ANOVA with Tukey’s multiple comparison test were used for statistical analysis. Error bars represent standard deviation (SD)

Free fatty acids have a well-documented role in obesity-associated endothelial dysfunction. To compare the signalling properties of different fatty acids we stimulated endothelial cells with the most common fatty acids in human serum with different chain length and saturation. In initial tests to determine optimal concentration (Fig. S1A and 1B) we selected 150 µM concentration of fatty acids coupled to BSA as optimal concentration to induce maximum effects on formation of lipid droplets and stress associated signalling pathways. This concentration is within the range of serum FFA concentration reported in previous studies [20–22]. We found that only oleic acid (OA, cis C18:1 (n-9)) efficiently induced formation of lipid droplets which were stained using BODIPY 493/305 dye (Fig. 1A and B). In contrast, saturated acids palmitate (PA) and stearate (SA) did not enhance the formation of lipid droplets, but they did promote accumulation of lipidated LC3B form (Fig. S1B and 1 C) and phosphorylation of the stress associated kinases p38 and JNK1/2.

**Fig. 2:**
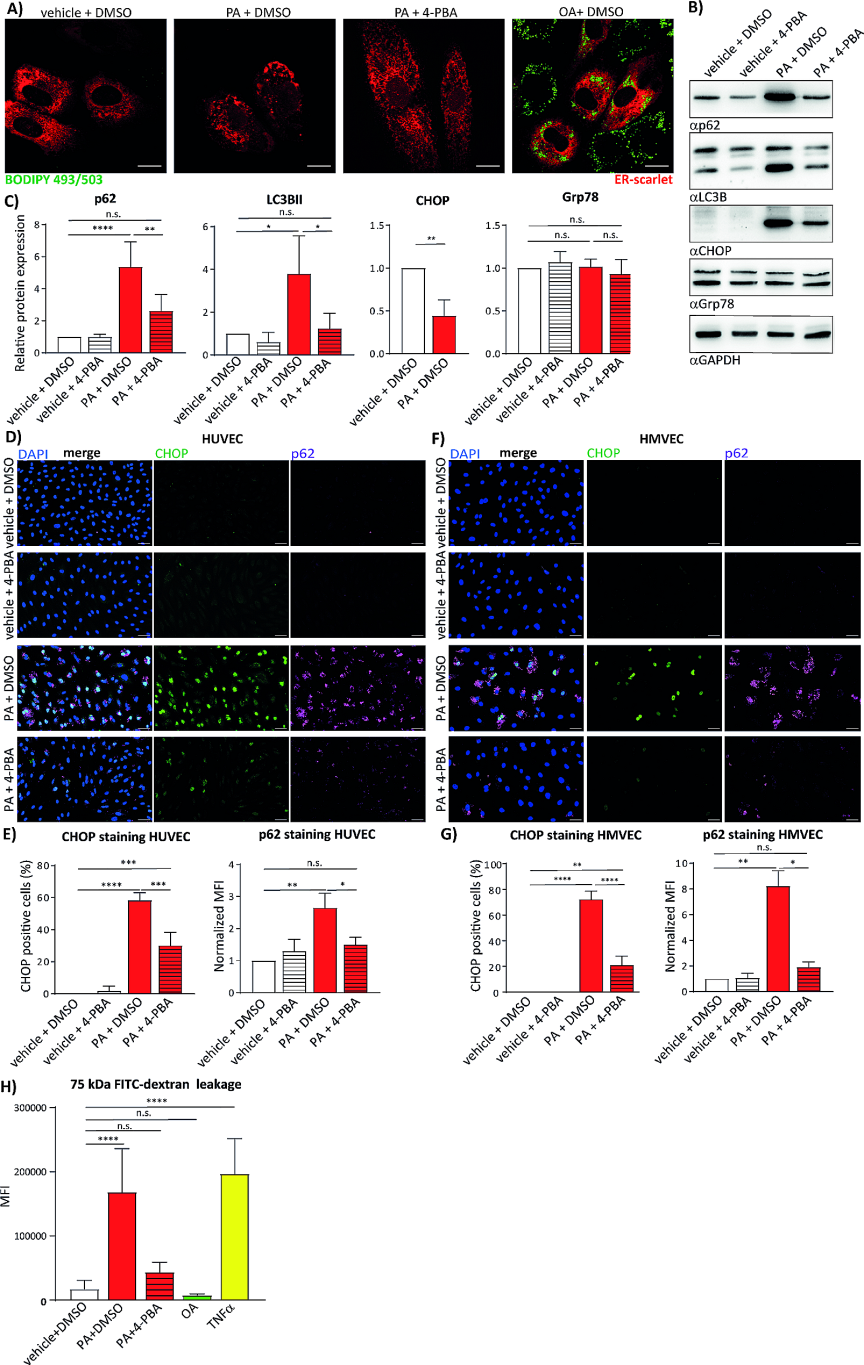
ER stress inhibition restores palmitate-dependent impairment of autophagy and induction of UPR in macro- and microvascular endothelial cells. (**a**) Immunofluorescence of the ER morphology in HUVEC treated with 150 μm of oleate (OA) or palmitate (PA) in combination with DMSO or 2.5 mM 4-PBA. Prior to treatment the cells were transfected with the ER-scarlet probe. Lipid droplets were stained using BODIPY 493/503. The samples were imaged using Zeiss AxioObserver. Scale bar represents 15 μm. **(b)** Immunoblot analysis of HUVEC treated for 16 h with 150 μm PA in combination with DMSO or 2.5 mM 4-PBA. Cells were lysed in 2x sample buffer and analysed for expression of LC3B, p62, CHOP and Grp78. GAPDH immunblot was used as a loading control. **(c)** Quantification of immunoblot results from (b). 20 images per condition were analyzed in total in 3 independent experiments. **(d)** Immunoflourescence analysis of CHOP and p62 expression in HUVEC. The cells were treated as in (**b**), fixed and stained with anti-CHOP and anti-p62 antibodies and DAPI. Scale bar represents 50 μm. **(e)** Quantification of immunofluorescence analysis depicted in (**d**). For CHOP quantification percentage of CHOP positive cells is displayed and for p62 mean fluorescence intensity (MFI) normalized to control sample. 5 images per condition were analyzed in each of 3 independent experiments. **(f)** Immunofluorescence analysis of CHOP and p62 expression in HMVEC. Sample preparation and imaging was performed as in (**d**). **(g)** Quantification of CHOP and p62 staining in HMVEC depicted in (**f**) was performed as described for HUVEC in (**e**). (**i**) FITC labelled dextran leakage assay was performed in HUVEC. Cells were seeded on Transwell inserts and treated for 16 h with BSA + DMSO as a control, 150 µM PA + DMSO or 150 µM PA + 2.5 mM 4-PBA, 150 µM OA or 10 ng/ml TNFa as positive control. The leakage of FITC-labelled 75 kDa Dextran into lower compartment was measured by reading of mean fluorescence intensity at 488 nm using the microplate reader. 3 technical replicates were measured for each condition in 3 independent experiments. GraphPad Prism software and one-way ANOVA with Tukey’s multiple comparison test were used for statistical analysis. Error bars represent standard deviation (SD)

Since oleate and palmitate in our experiments and also in published literature evoked profoundly different signalling effects on endothelial cells, and these two fatty acids are the most common in human serum, we decided to continue our experiments with oleate and palmitate. ER stress-induced UPR is one of the major cellular processes initiated by the excess of fatty acids, therefore we first analysed the morphology of the ER in endothelial cells upon treatment with fatty acids. As shown in Fig. S1D, palmitate treatment disrupted the ER network morphology, whereas oleate treatment did not. We compared next if efficient induction of lipids droplets by oleate and its sequestration in triglycerides protects the cells from eventual lipotoxic effects of oleate. We found that the inhibition of trilglyceride synthesis and of the subsequent lipid droplet formation by co-treatment with the diacylglcerol acyl transferase 1 (DGAT1) inhibitor A922500 and diacylglcerol acyl transferase 2 (DGAT2) inhibitor PF-06424439 did not disrupt the morphology of the ER (Fig. S1 F). To corroborate these findings, we compared oleate and palmitate induced signalling by analysing the UPR, autophagy markers and pp38 levels using immunofluorescence and western blotting. Microscopy images in Fig. 1C suggest that palmitate efficiently induces expression of UPR marker CHOP and oleate does not, even upon DGAT1 or DGAT2 inhibition. In contrast, palmitate induced UPR was further enhanced only upon DGAT1 and not upon DGAT2 inhibition as obvious in both microscopy images and western blot analysis (Fig. 1C, D and E). Compared to palmitate, oleate did not efficiently induce accumulation of the autophagy receptor p62, and the lipidated form of LC3B, whereas slight induction of the latter was observed upon DGAT1 inhibition in oleate treated cells (Fig. 1D and E). P38 phosporylation was as well enhanced only upon palmitate treatment and was not affected by oleate alone or in combination with DGAT1 or DGAT2 inhibitors (Fig. 1D and E). In order to test if pharmacological inhibition of the ER stress can revert palmitate induced signalling we co-treated HUVEC with the ER stress inhibitor 4-PBA and palmitate. Immunofluorescence pictures of the HUVEC transfected with the ER Scarlet fluorescent marker revealed that 4-PBA indeed partially reverts palmitate-induced disruption of the ER morphology (Fig. 2A). In addition, data obtained from Western blot analysis showed that 4-PBA co-treatment reduced the palmitate-induced accumulation of p62 and CHOP by 50% and 60%, respectively (Fig. 2B and C). In contrast to CHOP, levels of the UPR-associated chaperon Grp78 were not affected by palmitate treatment (Fig. 2C). Moreover, lipidated LC3B was restored to the control level in samples which were co-treated with 4-PBA (Fig. 2B and C). These data were corroborated by immunofluorescence staining of p62 and CHOP in HUVEC (Fig. 2D and E) and MVEC cells (Fig. 2F and G). 4-PBA reduced levels of both p62 and CHOP in HUVEC cells by 50%, whereas four-fold reduction of palmitate-induced accumulation of both markers was measured in MVEC cells (Fig. 2E and G). Finally, we tested if the disruption of the endothelial barrier by palmitate is restored upon 4-PBA co-treatment. Using 75 kDa FITC-labelled dextran leakage experiments, we found that palmitate induced the leakage of endothelial barrier comparable to TNFα, whereas oleate did not show any effect (Fig. 2H). Indeed, 4-PBA reduced the leakage of endothelial barrier caused by palmitate by approx. 75%. Interestingly, we did not observe enhanced expression of leukocyte adhesion receptor VCAM-1 upon treatment with palmitate (Fig. S1E), suggesting that palmitate did not induce pro-inflammatory response in our experiments. In accordance with these results, THP-1 cells did not adhere to endothelial cells pre-treated with palmitate in comparison to extensive adhesion observed in TNF-α-treated cells (Fig. S1 F). Together, these results suggest that the excess of saturated fatty acids impair autophagy in micro- and macrovascular endothelial cells via the ER-stress dependent mechanism.

### Levels of autophagy receptor p62 are elevated in serum of obese individuals

**Fig. 3:**
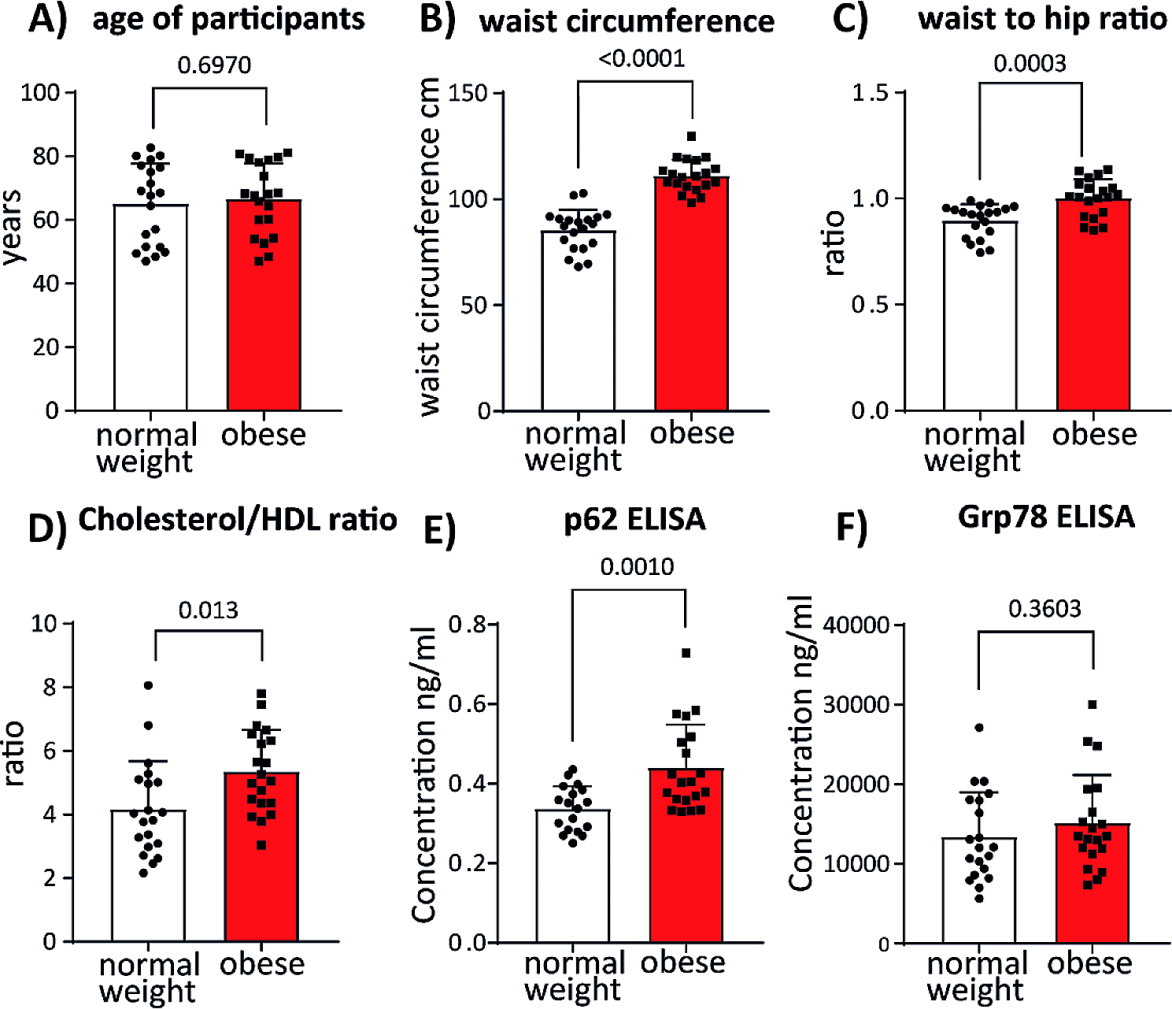
p62 levels are increased in serum of obese individuals. (**a**) Age of selected CARLA Study participants grouped in normal weight (BMI 18.5–24.99 kg/m2, *n* = 20) and obese individuals (BMI 30.00–34.99 kg/m2, *n* = 20). Waist circumference (cm) **(b)** and waist-to-hip-ratio **(c)** are depicted for normal weight and obese participants. **(d)** Cholesterol/HDL ratio in normal weight and obese sample groups is shown. Cholesterol and HDL concentrations were measured as described previously **(Greiser et al.,.).** Results of ELISA-based quantification of p62 **(e)** and Grp78 **(f)** in human serum. Each sample was measured in duplicate. Outliers were identified using GraphPad Prism and excluded from graphical representation and statistical analysis. Student’s t-test was performed to test for statistical difference between sample groups. Error bars represent standard deviation (SD)

Increased plasma levels of free fatty acids are characteristic for obesity and diabetes type 2. FFAs are released from adipose tissue, but can also originate from the dietary fat processing and inefficient uptake by adipose tissue [1]. To test if we can detect changes in levels of p62 and CHOP in vivo, we performed the ELISA analysis on sera obtained from participants of the CARLA study. Sera of 20 individuals with healthy body mass index (BMI 18.5–24.99 kg/m2) and 20 obese individuals (BMI 30.00–34.99 kg/m2) were analyzed for p62, CHOP and Grp78 expression. There was no difference in sex and age distribution (Fig. 3A) in both participant groups, and as expected waist to hip ratio and waist circumference were significantly increased in obese group (Fig. 3B and C). Furthermore, unhealthy metabolic profile was reflected in increased cholesterol/ HDL ratio in obese participants (4.16 vs. 5.36) (Fig. 3D). Using ELISA we found that p62 was increased from 0.34 ng/ml in serum of individuals with normal weight to 0.44 ng/ml in serum of obese individuals suggesting that the autophagy is disturbed by excess of fatty acids also in in vivo situation (Fig. 3E). Although we could not detect the UPR-associated transcription factor CHOP by ELISA technique in tested sera, we were able to quantify levels of UPR chaperone Grp78 in all samples. In these experiments, we did not find significant increase of Grp78 concentration in serum of obese individuals (Fig. 3F). These results are in agreement with our observation that Grp78 expression was not enhanced upon palmitate treatment in endothelial cells (Fig. 2C).

### ER stress inhibition enhances lipid droplet formation and restores palmitate-induced cholesterol synthesis

**Fig. 4:**
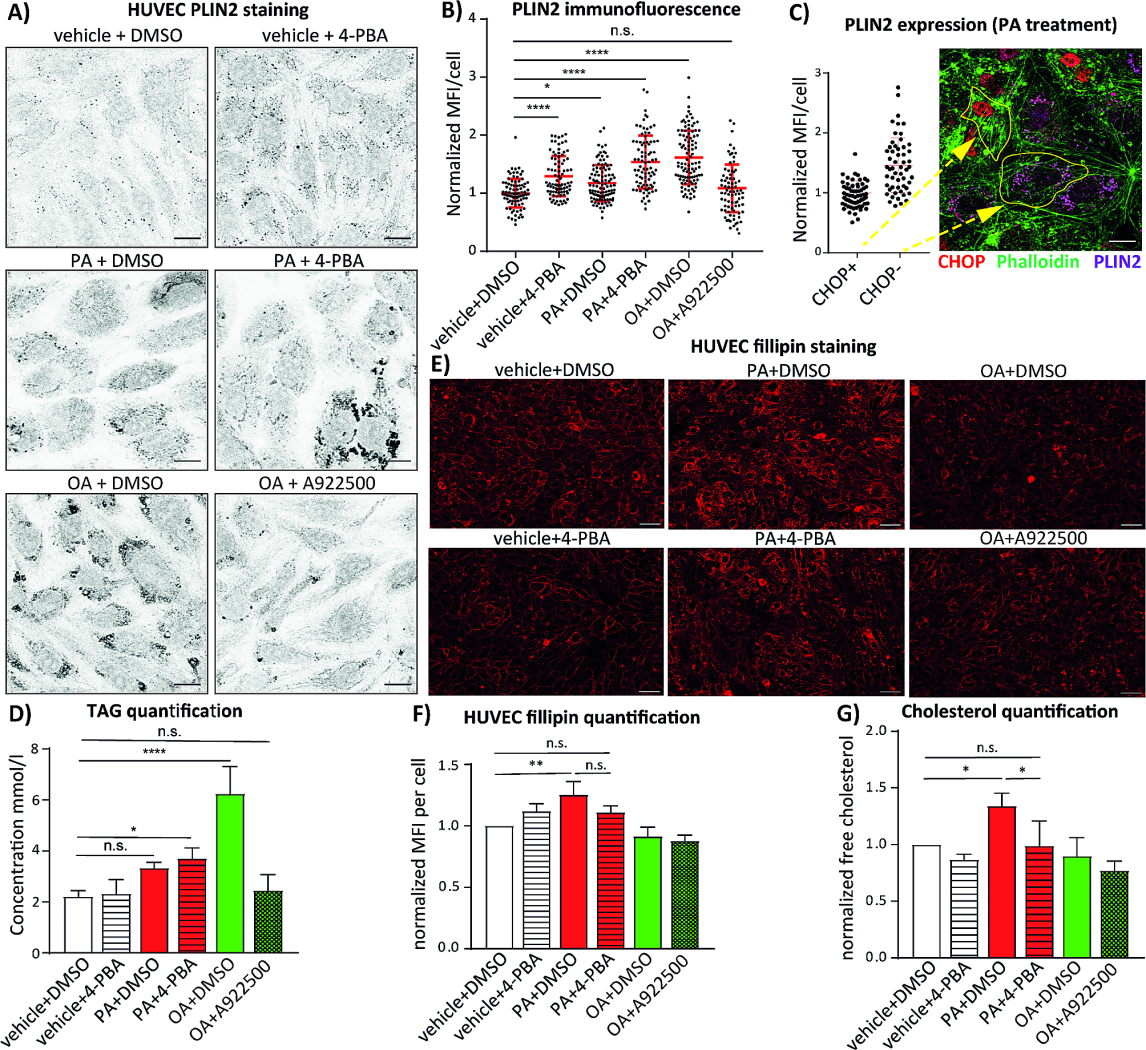
4-PBA co-treatment re-directs palmitate metabolism towards TG production and away from cholesterol synthesis. (**a**) Immunofluorescence analysis of PLIN2 expression in HUVEC treated with 150 µM palmitate (PA) alone or in combination with 2.5 mM 4-PBA for 16 h. As positive control the cells were treated with 150 µM oleate (OA) alone or in combination with 5 µM A922500. The samples were imaged using Zeiss AxioObserver. Scale bar represents 15 μm. **(b)** Quantification of PLIN2 staining from (**a**). Mean fluorescence intensity of PLIN2 was measured in 81–105 cells per condition in three independent experiments. Mean fluorescent intensity per cell normalized to control condition is shown in the graph. **(c)** Immunofluorescence staining of CHOP and PLIN2 in HUVEC treated with PA as in (**a**). CHOP is depicted in red, PLIN2 in magenta and actin staining in green. Scale bar represents 15 μm. Quantification of PLIN2 staining in CHOP positive (*n* = 67) and CHOP negative (*n* = 55) cells. Yellow arrows point towards representative CHOP^+^ and CHOP ^–^ cells. **(d)** Quantification of total triglycerides (TG) in extract of HUVEC treated as in (**a**). Data obtained from three independent experiments are shown. **(e)** Representative images of free cholesterol staining in HUVEC treated as in (**a**). After the treatment, the cells were fixed and stained with 25 ug/ml of Fillipin III for 30 min and immediately imaged using Zeiss AxioObserver. Scale bar represents 50 μm. **(f)** Quantification of the Fillipin III staining from (**e**). Normalized mean fluorescence intensity of Fillipin III per cell is depicted. 5 images per condition were analyzed in three independent experiments. **(g)** Measurement of free cholesterol content in HUVEC treated as in (**a**). Free cholesterol was quantified using Cholesterol/Cholesteryl Ester Assay Kit in three independent experiments. GraphPad Prism software and one-way ANOVA with Tukey’s multiple comparison test or Student’s t-test were used for statistical analysis. Error bars represent standard deviation (SD)

In previous experiments, we demonstrated that the ER stress inhibition restores negative effects of palmitate on autophagy and expression of the UPR markers in endothelial cells. We hypothesized that the pharmacological inhibition of the ER stress redirects palmitate-induced changes in lipid metabolism to deliver less disruptive lipid species. Although palmitate alone did not enhance the neutral lipid synthesis, as demonstrated by BODIPY staining (Fig. 1A and B), we tested if 4-PBA could modulate palmitate response towards storage lipid synthesis and lipid droplet formation, which would protect endothelial cells from lipotoxicity. Indeed, we found that palmitate in combination with 4-PBA significantly upregulated the levels of lipid droplet marker PLIN-2 to similar degree achieved upon treatment with oleate (Fig. 4A, B and Fig. S2 A). Remarkably, 4-PBA alone was more potent in induction of PLIN-2 expression than palmitate treatment. Our immunofluorescence analysis of PLIN-2 staining showed the large grade of heterogeneity in PLIN2 expression among cells after the treatment with palmitate. Upon closer inspection, we found that the cells, which expressed PLIN-2, did not express the UPR marker CHOP (Fig. 4C). Protective effects of 4-PBA resembled the ability of oleate to prevent palmitate-induced ER stress reported before [23]. Therefore, we tested if addition of oleate would prevent palmitate or stearate-induced CHOP expression in HUVEC. As shown in Fig. S2B and S2 C, oleate completely abrogated CHOP induction in both palmitate or stearate treated cells and restored lipid droplet formation in palmitate treated HUVEC. These data supported our hypothesis that the re-direction of palmitate-induced lipids towards storage lipids and lipid droplets might protect the cells from the ER stress. In following experiments, we studied the effects of 4-PBA on triglyceride synthesis (TG) rate in cells treated with palmitate. We found that palmitate in combination with 4-PBA increased the synthesis of TG two-fold in comparison to the vehicle control (Fig. 4D), whereas palmitate alone was not effective. Another line of evidence came from experiments performed with the lipin inhibitor propranolol. Similarly to DGAT1 inhibition (Fig. 1C, D and H), lipin inhibition further promoted palmitate induced UPR as well, suggesting that additional impairment of the residual palmitate-induced triglyceride synthesis is even more detrimental (Fig. S2 D and E).

Considering the well-documented role of saturated fatty acids in the synthesis of cholesterol and the increased cholesterol/HDL ratio in serum of obese CARLA Study participants (Fig. 3D), we analysed the cholesterol levels in HUVEC by Fillipin III staining and colorimetric quantification kit. As shown in Fig. 4E and F, palmitate treatment increased the intensity of the Fillipin III staining by 30% and 4-PBA restored this effect close to the control condition. Similar data were obtained by total cholesterol quantification, where palmitate increased the cholesterol concentration up to 40% in comparison to vehicle control and 4-PBA blocked this effect of palmitate efficiently (Fig. 4G). These data indicate that pharmacological inhibition of the palmitate-induced ER stress protects endothelial cells from lipotoxicity by diverting lipid metabolism towards TG synthesis and by reducing cholesterol synthesis.

### Ceramide synthesis is crucial for palmitate effects on autophagy, ER stress and cholesterol upregulation

**Fig. 5:**
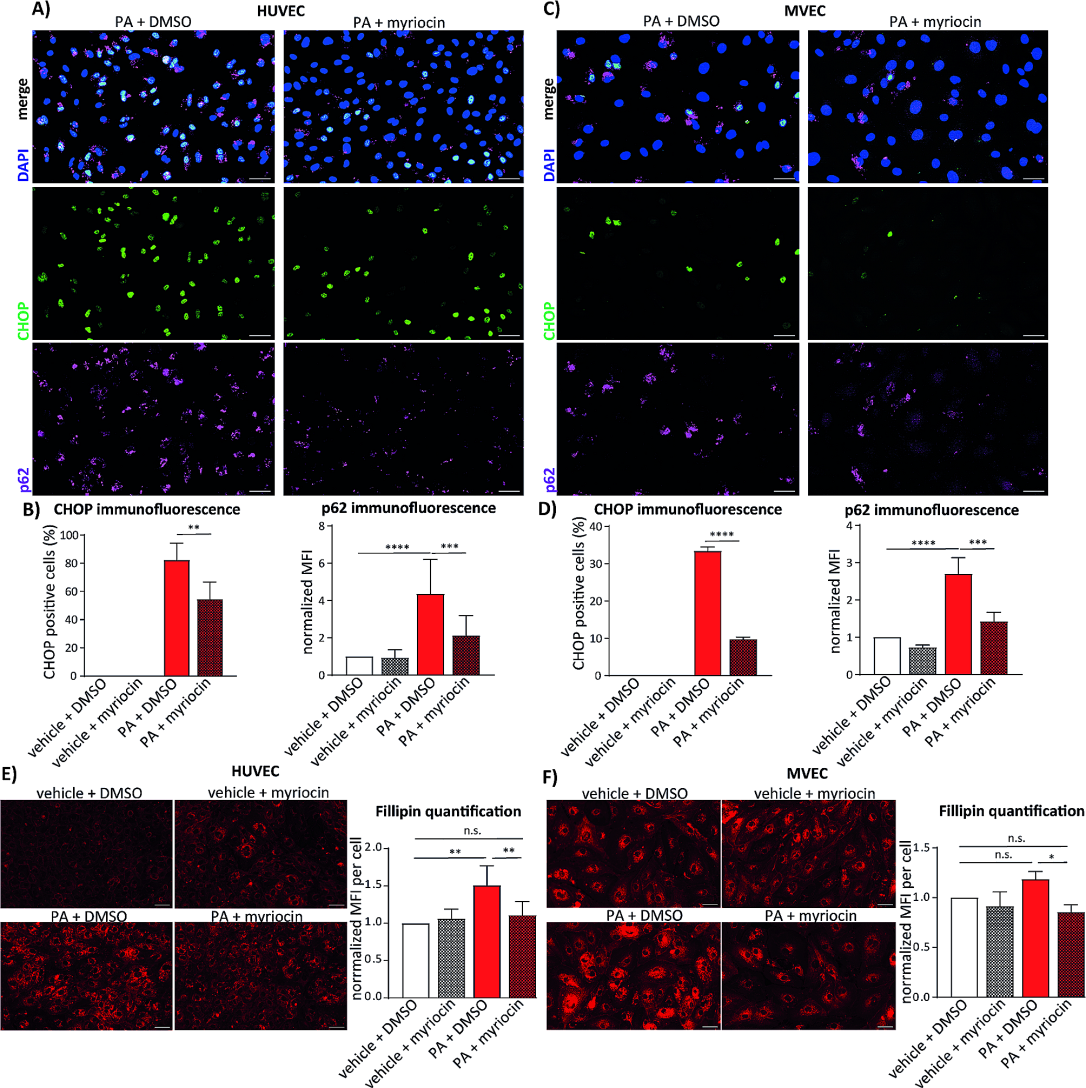
Inhibition of ceramide synthesis reverts effects of palmitate in micro- and macrovascular endothelial cells. (**a**) Immunoflourescence analysis of CHOP and p62 expression in HUVEC. The cells were pre-treated with 500 nM myriocin for 8 h and then for additional 16 h with 150 µM palmitate (PA). Afterwards the cells were fixed and stained with anti-CHOP and anti-p62 antibodies and DAPI. **(b)** Quantification of CHOP and p62 immunofluorescence analysis in HUVEC. Percentage of CHOP positive HUVEC is depicted on the left and normalized mean fluorescence intensity (MFI) of p62 signal is shown on the right side. 5 images were analyzed per condition in three independent experiments. **(c)** Immunoflourescence analysis of CHOP and p62 expression in MVEC. The samples were prepared as in (**a**). **(d)** Quantification of CHOP and p62 immunofluorescence analysis in MVEC. Percentage of CHOP positive HUVEC is depicted on the left and normalized mean fluorescence intensity (MFI) of p62 signal per cell is shown on the right side. 5 images were analyzed per condition in three independent experiments. **(e)** Representative images of free cholesterol staining in HUVEC treated as in (**a**). After the treatment, the cells were stained with 25 ug/ml of Fillipin III for 30 min and immediately imaged using Zeiss AxioObserver. On the right side quantification of the Fillipin III staining is shown. 5 images were analyzed per condition in three independent experiments. **(f)** Representative images of free cholesterol staining in MVEC were obtained and analysed as in (**e**). Scale bars in all images represent 50 μm. GraphPad Prism software and one-way ANOVA with Tukey’s multiple comparison test or Student’s t-test were used for statistical analysis. Error bars represent standard deviation (SD)

To disentangle lipid metabolism pathways required for palmitate lipotoxicity in endothelial cells we performed small-scale siRNA screen targeting all major membrane and storage lipid synthesis enzymes (Fig. S3 A). In this assay, we analysed the expression of p62 as most prominently induced protein marker upon palmitate treatment in our previous experiments. Although endothelial cell viability was affected by blockage of lipid metabolism, we found that only siRNAs targeting ceramide synthases 2, 5 or 6 reduced the number of p62 positive cells after palmitate treatment to 20%. In comparison, 40% of endothelial cells transfected with the non-targeting control siRNA showed p62 expression in immunofluorescence images (Fig. S3B). siRNA-mediated p62 knockdown efficiency was confirmed by both Western blot and immunofluorescence analysis (Fig. S3 C and D). Results of siRNA experiments suggested that ceramides might play a crucial role in palmitate-dependent autophagy impairment. Next, we tested if myriocin, the pharmacological inhibitor of the serine palmitoyl transferase, which catalyses the first step in ceramide synthesis, can rescue lipotoxic effects of palmitate on endothelial cells. As shown in Fig. 5A and B, myriocin diminshed both p62 and CHOP expression in palmitate-treated HUVEC. In parallel, we tested if microvascular endothelial cells MVEC respond in similar manner as macrovascular HUVEC. In MVEC myriocin was even more effective in reducing of the palmitate-dependent p62 and CHOP accumulation, to 30% and 50%, respectively (Figs. 4D and 5C). Taken together, these data confirm that ceramides are relevant for the palmitate-mediated autophagy block and ER stress induction.

In next set of experiments, we tested if the observed induction of the cholesterol synthesis by palmitate is also restored by the ceramide synthesis inhibition. Using Fillipin III staining we found that myriocin pre-treatment in both tested endothelial cell types HUVEC and MVEC, restored the free cholesterol levels to control condition (Fig. 5E and F). These results additionally underline the crucial role of ceramides in lipotoxic effects of palmitate on lipid metabolism in endothelial cells.

### Lipidomic analysis reveals robust increase in saturation of membrane phospholipids after palmitate treatment in endothelial cells

**Fig. 6:**
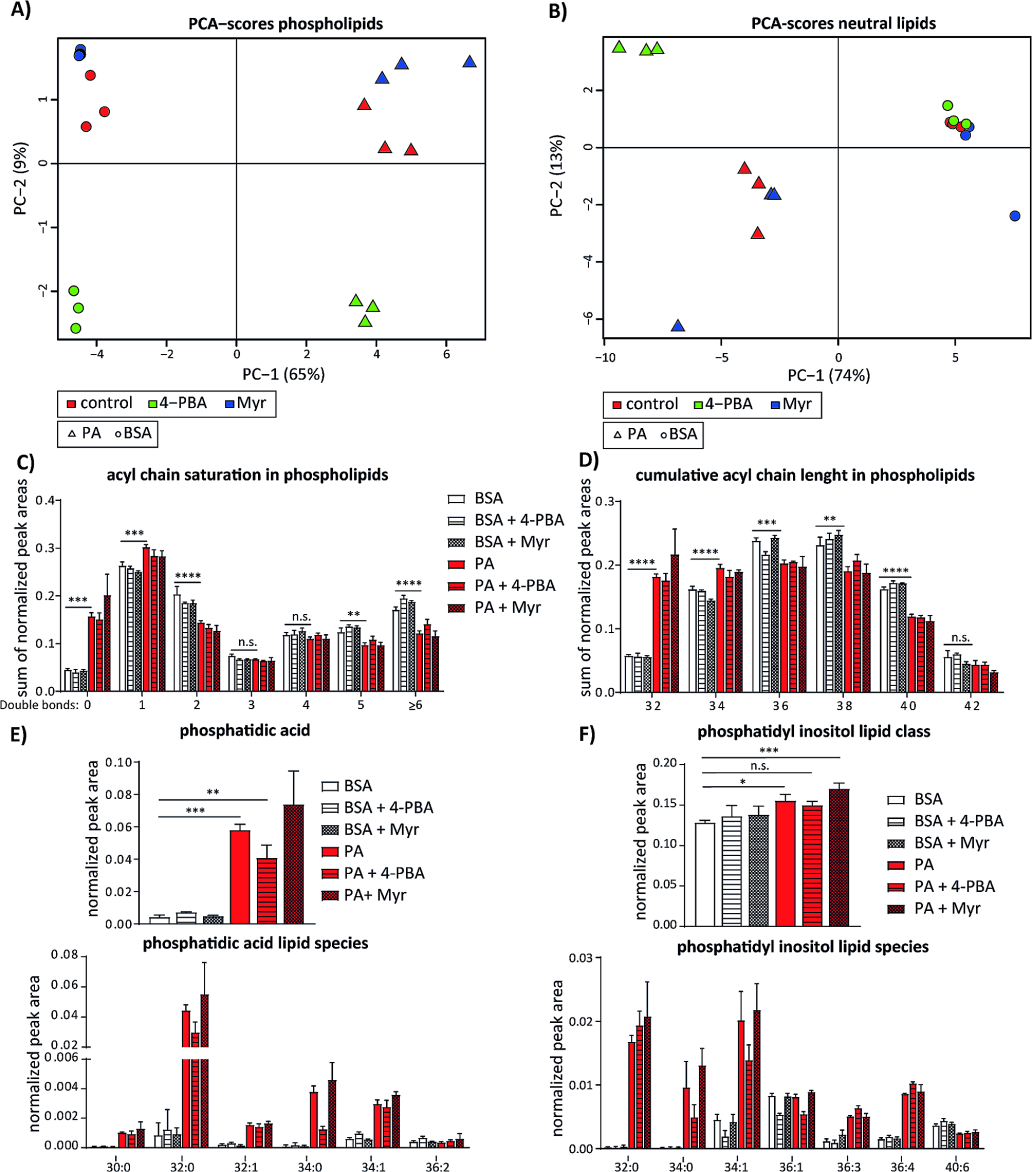
Palmitate induces synthesis of phosphatidic acid and enhances saturation of phospholipids in endothelial cells. (**a**) Principal component analysis (PCA) score plot of phospholipids measured in HUVEC treated with 150 μm palmitate (PA) alone or in combination with 2.5 mM 4-PBA or 500 nM myriocin. **(b)** PCA score plot of neutral lipid profiles obtained from HUVEC treated as in (a). **(c)** Acyl chain saturation in annotated phospholipids. Phospholipids were grouped according to the number of double bonds in their acyl chains. Within each group, the sums of normalized peak areas for each treatment condition are shown. **(d)** Acyl chain length in annotated phospholipids. Phospholipids were grouped according to the number of C atoms in their acyl chains. Within each group, the sums of normalized peak areas for each treatment condition are shown. **(e)** Normalized peak areas of total phosphatidic acid (PA) lipid class and most common annotated PA species (under). **(f)** Normalized peak areas of total phosphatydil inositol (PI) lipid class and most common annotated PI species (under). *n* = 3, GraphPad Prism software and one-way ANOVA with Tukey’s multiple comparison test were used for statistical analysis. Error bars represent standard deviation (SD)

In above described experiments we studied the effects of palmitate on cell signalling and lipid metabolism in endothelial cells using various technical approaches. In order to perform a more systematic analysis of lipid metabolism, we performed mass spectrometry (MS)-based lipidomic analysis on HUVEC treated with palmitate in combination with the ER stress inhibitor 4-PBA or serin palmitoyil transferase inhibitor myriocin. The identification and relative quantification of phospholipids and neutral lipids was performed on 6 sample groups: vehicle, vehicle + 4-PBA, vehicle + myriocin, palmitate, palmitate + 4-PBA and palmitate + myriocin. The PCA analysis score plot of both phospholipids and neutral lipids (Fig. 6A and B) was generated to show the clustering trends among analysed sample groups. In case of phospholipids, the score plot represents 74% of total variance with principal component 1 contributing 65% and principal component 29%. As shown in Fig. 6A, all experimental groups separated from each other in the score plot of the phospholipid profiles. Palmitate-treated groups scattered at the right part of the plot and vehicle (BSA) groups at the left part. Furthermore, 4-PBA treated samples clustered at the lower part of the graph, whereas control and myriocin treated samples were scattered at the upper part of the plot. In case of neutral lipids, the score plot represents 87% of the total variance, including principal component 1 with palmitate-treated samples clustered at the right side of the PCA analysis score plot, whereas vehicle treated samples remained scattered at the right part. Co-treatment with inhibitors only separated 4-PBA and palmitate treated group from rest of samples exposed to palmitate. However, this effect of 4-PBA was not obvious in vehicle treated samples.

After performing identification and relative quantification of annotated phospholipids, we compared saturation level and total acyl chain length of membrane lipids in different experimental groups. Cumulative levels of all phospholipid species with 0, 1, 2, 3, 4, 5 and 6 and more double bonds are shown in Fig. 6C. Here we found that palmitate treatment induced 3-fold increase in relative amount of saturated phospholipids (0 double bonds) and phospholipids with one double bond in their acyl chains. Simultaneously, polyunsaturated phospholipids with 2, 5 or 6 and more double bonds were significantly decreased after palmitate treatment. These results suggest that palmitate dramatically increases saturation degree of membrane phospholipids. Furthermore, neither 4-PBA nor myriocin co-treatment showed significant effect on saturation of phospholipid acyl chains (Fig. 6C). In addition to saturation, we also compared abundance of membrane phospholipids with defined total length of acyl chains in our experimental groups. According to the total number of the C atoms in the acyl chains we separated lipids into the most common C32, C34, C36, C38, C40 and C42 groups. We found that that palmitate treatment significantly increased levels of C32 and C34 lipids, 3 fold and 20% respectively, whereas the relative amount of lipids with longer acyl chains C36, C38, C40 and C42 was reduced after palmitate treatment (Fig. 6D). Similar to acyl chains saturation, co-treatment with 4-PBA or myriocin did not significantly affect the length of the phospholipid acyl chains (Fig. 6D).

Palmitate treatment drastically increased the relative abundance of phosphatidate (PtdOH) and phosphatidyl inositol (PI) was 10% increased under same conditions (Fig. 6E and F). As shown in Fig. 6E, relative amount of PtdOH after palmitate treatment was increased 14 fold to almost 6% of total annotated phospholipids in this sample group. Co-treatment of HUVEC with 4-PBA decreased the palmitate-induced levels of PtdOH for 30%, which might suggest that 4-PBA either blocks the PtdOH synthesis or promotes its downstream processing towards phospholipids (PLs) or TG synthesis. None of other major membrane phospholipid classes were significantly changed due to palmitate treatment. Closer inspection of levels of specific types of PtdOH and PI revealed a drastic change in saturation pattern of these phospholipid types. A shown in Fig. 6E in comparison to vehicle control palmitate induced a strong increase in PI species with high saturation degree such as PtdOH 30:0, 32:0, 32:1, 34:0 and 34:1. Although Myrocin co-treatment did not show any effect on PtdOH levels, 4-PBA co-treatment hindered palmitate-dependent increase in PtdOH 32:0 and 34:0 lipid types. In similar manner, levels of PI 32:0 (70 fold), PI 34:0 (74 fold) and PI 34:1 and also of certain PI types with unsaturated acyl chains such as PI 40:6 were increased upon palmitate treatment. 4-PBA again reverted palmitate-enhanced increase in PI 34:0 and 34:1 lipids. In brief, these results show that palmitate treatment changes the composition and saturation of phospholipids in endothelial cells, which might induce changes in membrane architecture in these cells. Furthermore, 4-PBA, unlike myriocin, was able to restore the levels of total PtdOH and of certain saturated phospholipid species, suggesting that the ER stress is crucial signalling event to potentiate and integrate palmitate-induced changes in membrane composition and lipid metabolism.

### ER stress inhibition relieves stalling in TG synthesis pathway induced by palmitate

**Fig. 7:**
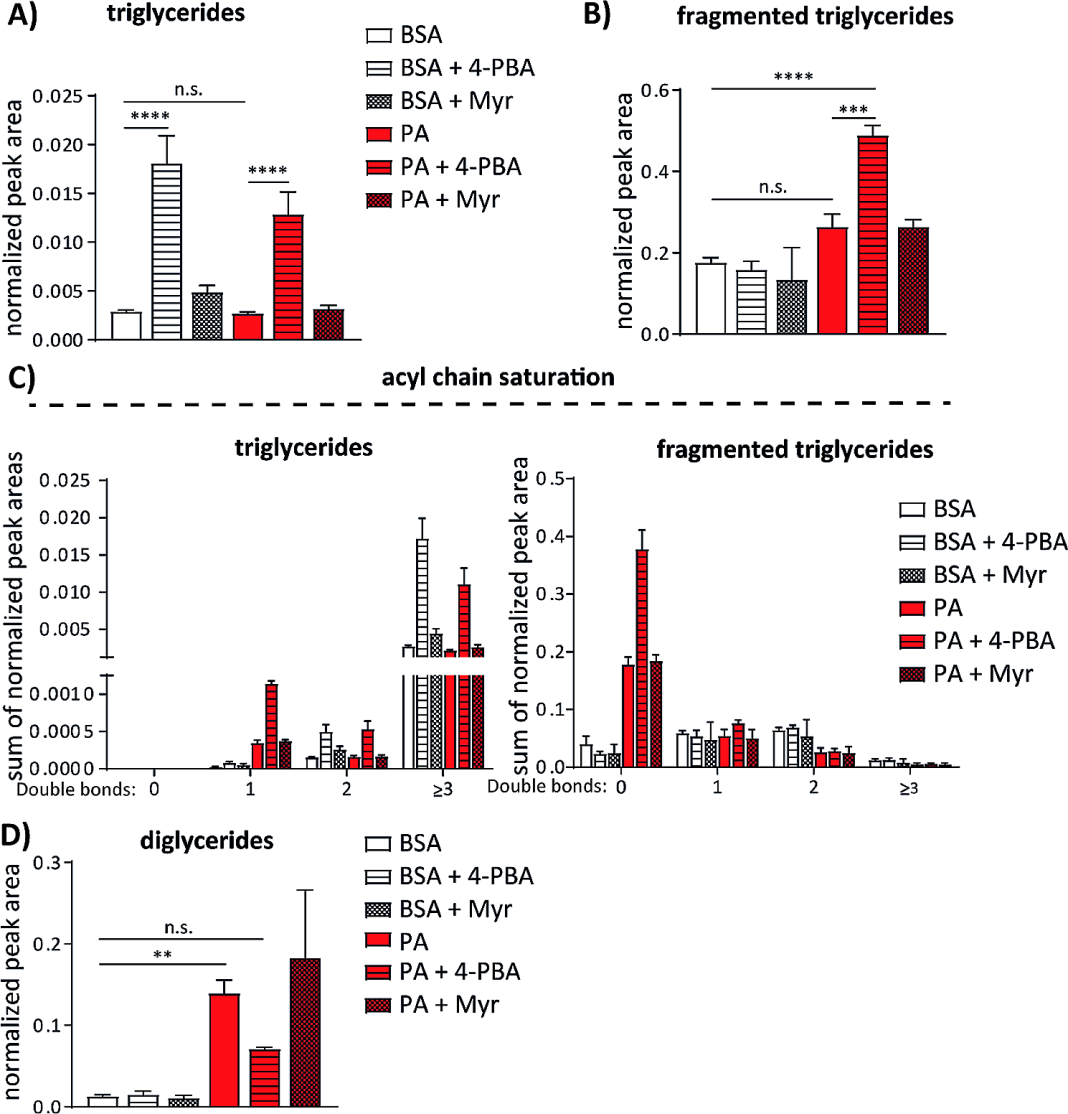
ER stress inhibition switches palmitate metabolism in endothelial cells towards formation of TGs with saturated acyl chains. (**a**) Normalized peak areas of triglyceride (TG) fraction detected by mass spectrometry in HUVEC treated for 16 h with 150 μm palmitate (PA) alone or in combination with 2.5 mM 4-PBA or 500 nM myriocin. **(b)** Normalized peak areas of fragmented TG fraction detected in HUVEC treated as in (**(a)**). **(c)** Analysis of the acyl chain saturation in TG and fragmented TG fraction. TG and fragmented TG species were grouped according to the number of double bonds in their acyl chains. Within each group, the sums of normalized peak areas for each treatment condition are shown. **(d)** Normalized peak areas of fragmented TG fraction detected in HUVEC treated as in (**a**). *n* = 3, GraphPad Prism software and one-way ANOVA with Tukey’s multiple comparison test were used for statistical analysis. Error bars represent standard deviation (SD)

In order to decipher why palmitate is not efficiently promoting the synthesis of TG and formation of lipid droplets in endothelial cells (Figs. 1A and B and 4A, B and D) we also compared the relative amounts of TGs in our lipidomic analysis data. In accordance with the data described above, lipidomic analysis did not show increased amounts of TG in palmitate treated HUVEC (Fig. 7A). Only a slight increase in fragmented TG species, which are produced due to harsh ionization procedure during samples preparation for the mass spectrometry analysis, was detected after palmitate treatment (Fig. 7B). Surprisingly, treatment with 4-PBA alone enhanced the synthesis of TG 6 fold in comparison to vehicle control (Fig. 7A). 4-PBA in combination with palmitate also enhanced TG synthesis 5 fold in comparison to sole palmitate treatment. In case of fragmented TG species, only co-treatment with 4-PBA in combination with palmitate efficiently elevated the levels of fragmented TG when compared to vehicle control or palmitate treatment alone (Fig. 7B). These data suggested that 4-PBA increases TG synthesis rate and could thereby protect endothelial cells from palmitate treatment. Analysis of the acyl chains saturation in TG and fragmented TG species showed that TGs with fully saturated fatty acids were detected only in fragmented TG fraction (Fig. 7C). Their levels were increased several fold after palmitate treatment and further doubled in 4-PBA co-treatment group in comparison to only palmitate treated group. Levels of TG with unsaturated fatty acids did not significantly change in the fragmented TG fraction, but were increased in TG fraction after 4-PBA treatment alone or in combination with palmitate. These data suggest that 4-PBA alone enhances formation of TGs with unsaturated fatty acid chains, while the synthesis is shifted towards TGs with saturated fatty acids when 4-PBA is added in combination with palmitate (Fig. 7C).

Since levels of TG precursor PtdOH were strongly increased in palmitate-treated cells (Fig. 6E) and TG levels were not affected significantly under same conditions (Fig. 7A), we next compared the levels of diglyceride (DG), a TG precursor downstream of PtdOH. Lipidomic analysis revealed that also levels of DG were 11 fold increased upon addition of the palmitate to the cell culture medium (Fig. 7D). In these samples, DG levels reached almost 14% of total annotated lipids in the neutral lipid fraction. Co-treatment with 4-PBA also strongly reduced the levels of palmitate-induced DG (Fig. 7D), whereas co-treatment with myriocin did not show consistent effects on DG synthesis. Altogether, our data suggest that palmitate excess leads to blockade in TG synthesis pathway, resulting in accumulation of the precursor lipids PtdOH and DG. Moreover, 4–PBA co-treatment seems to relieve this palmitate induced blockade, by promoting the synthesis of TG and simultaneously decreasing the levels of PtdOH and DG.

### Palmitate-enhanced ceramide synthesis is restored to control levels upon ER stress inhibition

**Fig. 8:**
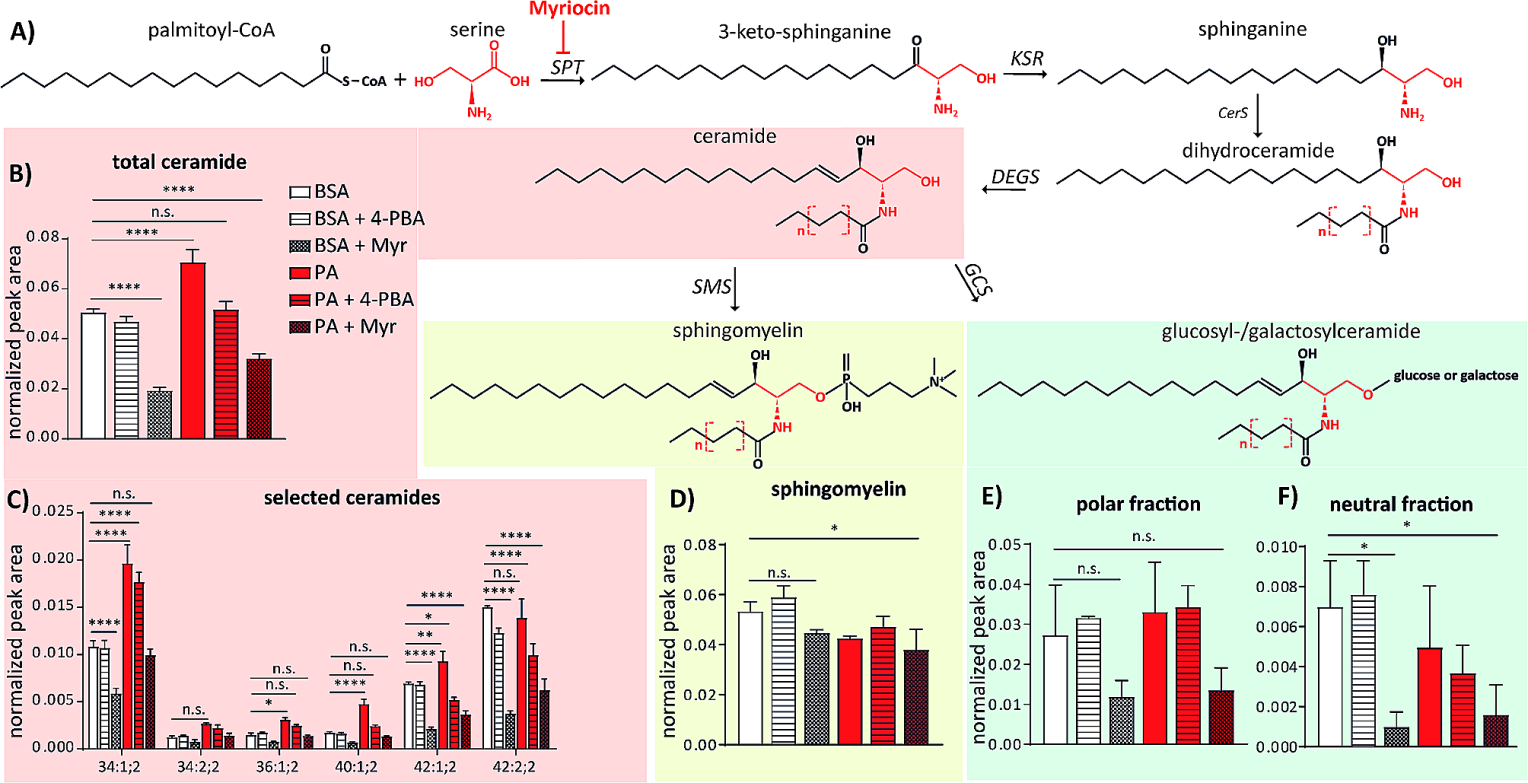
Induction of ceramide synthesis by palmitate in endothelial cells is blocked by 4-PBA. **a**) Scheme of the sphingolipid synthesis pathway. Serine palmitoyltransferase (SPT), 3-ketodihydrosphingosine reductase (KSR), ceramide synthase (Cers), sphingolipid delta-4 desaturase (DEGS), sphingomyelin synthase (SMS) and glucosylceramide synthase (GCS). (**b**) Normalized peak areas of total ceramides detected by mass spectrometry in HUVEC treated for 16 h with 150 μm palmitate (PA) alone or in combination with 2.5 mM 4-PBA or 500 nM myriocin. (**c**) Normalized peak areas of most abundant ceramide species detected by lipidomic analysis. (**d**) Normalized peak areas of sphingomyelin lipid class detected by lipidomic analysis. (**e**) Normalized peak areas of glucosyl- and galactosylceramides detected in polar (left) or neutral (right side) lipid fraction. *n* = 3. GraphPad Prism software and one-way ANOVA with Tukey’s multiple comparison test were used for statistical analysis. Error bars represent standard deviation (SD)

As shown in Fig. 4 and Fig. S3 inhibition of ceramide synthesis reduced the adverse effects of palmitate on lipid metabolism. To corroborate these findings we analysed the effects of palmitate on levels of ceramides and the downstream products of their metabolism, sphingomyelin and glucosyl-/galactosylceramide, using lipidomic analysis (Fig. 8A). In vehicle condition, relative amount of ceramides reached 5% of annotated neutral lipid fraction and it was reduced to 2% after pre-treatment with myriocin (Fig. 8B). In contrast, addition of palmitate increased relative ceramide levels to 7%. This increase did not occur in cells that were pre-treated with myriocin prior to addition of palmitate. Furthermore, co-treatment with 4-PBA also restored total ceramide levels to vehicle control condition (Fig. 8B); suggesting that the ER stress is crucial event for majority of palmitate-induced disturbances in lipid metabolism in endothelial cells. More detailed analysis of single ceramide species revealed that palmitate efficiently induced the synthesis of saturated ceramide types such as Cer 34:1;2, Cer 36:1;2 and 40:2;2, without obvious effect on ceramides containing very long fatty acids e.g. Cer 42:2;2 (Fig. 8C). As opposed to ceramide levels, relative amount of sphingomyelin was decreased upon palmitate addition to similar level as in samples treated with myriocin (Fig. 8D). In addition, palmitate did not significantly affect levels of glucosyl- or galactosylceramides (Fig. 8E). These data suggest that among other adverse effects on lipid metabolism, the excess of palmitate also directly enhances the production of ceramides in endothelial cells. High levels of ceramides in turn promote the ER stress response, which blocks the flux towards the downstream products of ceramide metabolism.

## Discussion

Endothelial activation and dysfunction are hallmarks of obesity, which affects over 25% of the population in Europe (WHO). While classically used parameters of obesity, such as body mass index (BMI), waist circumference and waist to hip ratio, are clearly correlated with the incidence of myocardial infarction [24], BMI, except in extreme cases, has not proven to be a useful parameter for prediction of the outcome of other CVDs, such as heart failure [25]. Therefore, it is conceivable that the composition and distribution of adipose tissue, and the type of lipids released into the circulation, may be far more relevant for the development of endothelial dysfunction and cardiovascular disease, as compared with the gross increase in body mass. Increased plasma levels of non-esterified fatty acids, also called free fatty acids (FFA), are characteristic for obesity and diabetes type 2. FFAs are released from adipose tissue, but can also originate from dietary fat processing and inefficient uptake by adipose tissue [1]. In cell culture experiments palmitate and products from the lipolysis of triglyceride-rich lipoproteins can directly impair endothelial barrier integrity in microvascular endothelial cells and human aortic endothelial cells [7, 8]. In vivo studies in mice have also shown that palmitate impairs endothelial function, by activating TLR signaling in endothelial cells, thus triggering an upregulation of leukocyte adhesion receptors, production of inflammatory cytokines and impaired glucose metabolism [12]. However, later studies in macrophages have shown that palmitate does not directly activate TLR, but it rather impairs the lipid metabolism in cells that were primed by TLR activation via another palmitate independent mechanism [13]. In order to decipher the molecular mechanisms underlying endothelial damage induced by palmitate we first compared the cellular response to fatty acids of varying chain length and saturation degree. In agreement with the work of Kuo at al. in 2017 we found that unsaturated fatty acids oleate and to lesser extent DHA induced formation of lipid droplets in endothelial cells, which was DGAT1 dependent. Furthermore, similar to data from mouse lung endothelial cells [23], we did not detect lipid droplets in HUVEC treated with the same concentration of saturated fatty acids palmitate or stearate. These results suggest that the exogenously added fatty acids follow different metabolic routes in endothelial cells depending on their saturation degree.

As lipid droplets are considered to have a protective role against lipotoxicity in endothelial and other cell types [23, 26], we compared if this was relevant for both saturated and unsaturated fatty acids in endothelial cells. As a readout for FFA-induced signaling, we studied the impact of diverse FFA types on ER stress-induced unfolded protein response (UPR) and autophagy, two cellular events with documented impact of saturated fatty acids in published literature [14]. In these experiments, we found that markers of the UPR pathway and autophagy were strongly induced when endothelial cells were treated with palmitate. However, this was not the case when oleate was used for the treatment, even in combination with the DGAT1 inhibition. These results suggest that the saturation degree of the long-chain fatty acids is crucial determinant for the activation of lipotoxic signaling events and also for their processing towards storage lipid forms in endothelial cells, similarly to previously described findings in other cell types [27]. Furthermore, the effects of palmitate were even more detrimental when the DGAT1 was simultaneously inhibited, suggesting that residual amount of palmitate is stored in triglycerides (TG) within lipid droplets. In contrast, DGAT2 inhibition did not affect palmitate-induced signaling, e.g. induction of CHOP expression. In accordance with the work of Kuo et al., 2017, these data confirm that DGAT1 is the more relevant isoform for the triglyceride synthesis in endothelial cells.

The ER is the major site of the cellular lipid metabolism where exogenously added fatty acids are directed towards formation of e.g. phospholipids or storage lipids. Furthermore, sFFA-induced ER stress and UPR affects the cellular metabolism at many levels such as protein translation, apoptosis or autophagy. In previous studies, it was demonstrated that palmitate inhibits autophagy flux upon prolongued exposure in multiple human cell types, including human aortic endothelial cells [28–30]. In accordance with these results, our data suggest that palmitate strongly inhibits autophagy flux in both micro- and macrovascular endothelial cells upon overnight incubation. Additionally, we found that co-application of the pharmacological ER-stress inhibitor 4-PBA significantly reverted the palmitate-induced ER morphology disruption, UPR signaling and the inhibition of the autophagy flux. This suggests that detrimental effects of sFFA on endothelial cells largely depend on the downstream metabolism of these fatty acids in the ER. This was further demonstrated by the protective effect of 4-PBA on palmitate–dependent loss of endothelial barrier integrity. Interestingly, protein folding-independent effects of 4-PBA on suppression of the UPR response were previously described in yeast [31] and ER stress inhibitors were shown to restore the ER membrane fluidity in mice [32] and reduce lipid accumulation in hepatoma cell lines [33].

Our initial data on palmitate-induced signaling events were obtained from in vitro experiments in primary human endothelial cells lines, however we were intrigued by massive increase in expression of the autophagy marker p62 and the UPR-related transcription factor CHOP and wondered if these changes were reproducible in more complex system. To test this hypothesis, we analyzed expression of p62, CHOP and Grp78 in sera of the participants of the epidemiological population-based cohort CARdiovascular Disease, Living and Ageing in Halle (*CARLA*) study [17]. Although we could not reliably detect CHOP in human serum using ELISA, p62 analysis showed significant increase in obese individuals (440 ng/ml) when compared to normal weight participants (337 ng/ml). Almost identical concentration of p62 (340 ng/ml) was reported in the control group sera in non-alcoholic fatty liver disease study by Wang et al. in 2018 [34]. To our knowledge, our findings are the first indication that p62 is detectably increased in serum of obese individuals. In contrast to p62, we did not find an increase in Grp78 levels in serum of obese individuals. Although the origin of p62 in serum might be non-endothelial, increased level of p62 in serum of obese individuals is a good indication that the autophagy flux is perturbed in obese individuals. While these data should be reproduced in a larger study in combination with determination of FFA profile in analyzed sera, our ELISA results do complement the findings from our cell culture experiments. In following experiments, we made effort to disentangle mechanistic background of palmitate induced ER stress response and the relevance of lipid droplet formation for this process. Lipid droplet formation was shown to be induced by the UPR itself and was identified as protective mechanism to relieve the ER stress, restore lipid metabolism and regulate autophagy in cells exposed to excess lipid concentrations [35–37]. In accordance with our BODIPY staining and previously published work on cardiomyocytes [27] palmitate did not enhance synthesis of the lipid droplet marker PLIN2. Only upon co-treatment with the ER stress inhibitor was the increase in lipid droplet formation detectable. Analysis of the TG levels, as major storage lipid components of the lipid droplets, showed that palmitate, unlike oleate, was ineffectively inducing TG synthesis similar to findings of Bosma et al. Furthermore, simultaneous inhibition of the ER stress, together with palmitate treatment, did induce TG synthesis. These data suggest that the ER malfunction induced by the metabolism of the excess palmitate is causative for ineffective formation of protective lipid droplets in endothelial cells.

In contrast to the effect on TG synthesis, palmitate treatment increased cholesterol synthesis in many investigated cell types and tissues [38]. Accordingly, we found that palmitate also enhanced cholesterol synthesis in endothelial cells and this increase also was reverted upon ER-stress inhibition using 4-PBA. While enhanced cholesterol synthesis might partially account for detrimental effects of palmitate on endothelial cell function, the effects of 4-PBA indicate that other lipid metabolites of palmitate might be responsible for its effects. To identify the lipid metabolites behind palmitate effects on endothelial cells, we performed a small-targeted siRNA screen and lipidomic analysis in HUVEC treated with palmitate or controls. In the first approach, we depleted 27 genes crucial for synthesis of all major phospholipid or storage lipid species and analysed the effects on palmitate-induced p62 expression as one of the most strongly induced markers inabove described experiments. Although, depletion of major genes involved in lipid metabolism was challenging in HUVEC, our siRNA screen analysis screen suggested that ceramide synthases 2, 4 and 6 and thereby ceramides or their metabolites might be the lipid species responsible for detrimental palmitate effects in endothelial cells. This is in accordance with previous observations that the excess of free fatty acids induces synthesis of sphingolipids in order to prevent the membrane damage by free fatty acids [39]. The important role of sphingolipids in endothelial cell response to palmitate was further supported by pharmacological inhibition of the serine palmitoyl transferase, an enzyme catalyzing the first reaction step in ceramide synthesis. Namely, inhibition of serine palmitoyl transferase by myriocin could reduce expression of palmitate-induced p62 and CHOP and revert cholesterol levels to control condition in our experiments in micro- and macrovascular endothelial cells. The role of ceramides in development in cardiovascular disease (CVD) and their use as biomarkers for prediction of the CVD outcome has gained the attention of scientific community in the past decade [40]. Especially ceramide species containing C16, C18, and C24:1 in their acyl chains proved to be usefull and even better predictive marker for fatal CVD outcome than LDL [41]. To systematically identify specific ceramide and other lipid species induced by palmitate in endothelial cells we performed mass spectrometry-based lipidomic analysis in HUVEC treated with palmitate alone or in combination with 4-PBA or myriocin. While palmitate treatment did dramatically increase the amount of PtdOH in HUVEC and showed milder effects on PI abundance, relative amount of other phospholipid classses was not affetced by palmitate. However, in all phospholipid types addition of palmitate had a profund impact on architecture of the acyl chains within phospholipids molecules. The most prominent effect we found was increased saturation in phospholipid acyl chains with accompanying decrease in abundance of polyunsaturated fatty acids within phospholipids. This increase in saturation was strongest within PtdOH and PI phospholipid class. Similar to our results in endothelial cells, Volmer et al. found that increased saturation of membrane lipids induced UPR response in mouse fibroblast, independently of protein folding within the ER [14]. Interestingly, ER stress inhbition with 4-PBA partially hindered palmitate-dependent increase in saturated PtdOH and PI lipid types. Furthermore, our lipidomic analysis showed that total acyl chain lenght in phospholipids also was switched towards phospholipids with C32 or C34 upon palmitate treatment, with simultaneous decrease in longer acyl chains with C ≥ 36. Similar impairment of phospholipid metabolism due to palmitate incorporation in phospholipids leading to UPR response was previously reported in hepatocytes [42]. Surprisingly, neither 4-PBA nor myriocin showed any significant effect on acyl chain saturation or chain lenght of total phospholipids when applied alone or in co-treatment with palmitate. Previously, 4-PBA was shown to increase the ER membrane fluidity in fungus-induced asthma model in mouse lungs and human bronchial epithelial cells [32] suggesting a potential mechanistic explanation for beneficial effects of 4-PBA on UPR activation. Although ameliorating effects of 4-PBA on palmitate-induced UPR, autophagy flux and cholesterol synthesis could be attributed to partially restored levels of PtdOH or restored saturation of certain phospholipid species, beneficial effects of myriocin cannot be explained by effects on phospholipids.

**Fig. 9:**
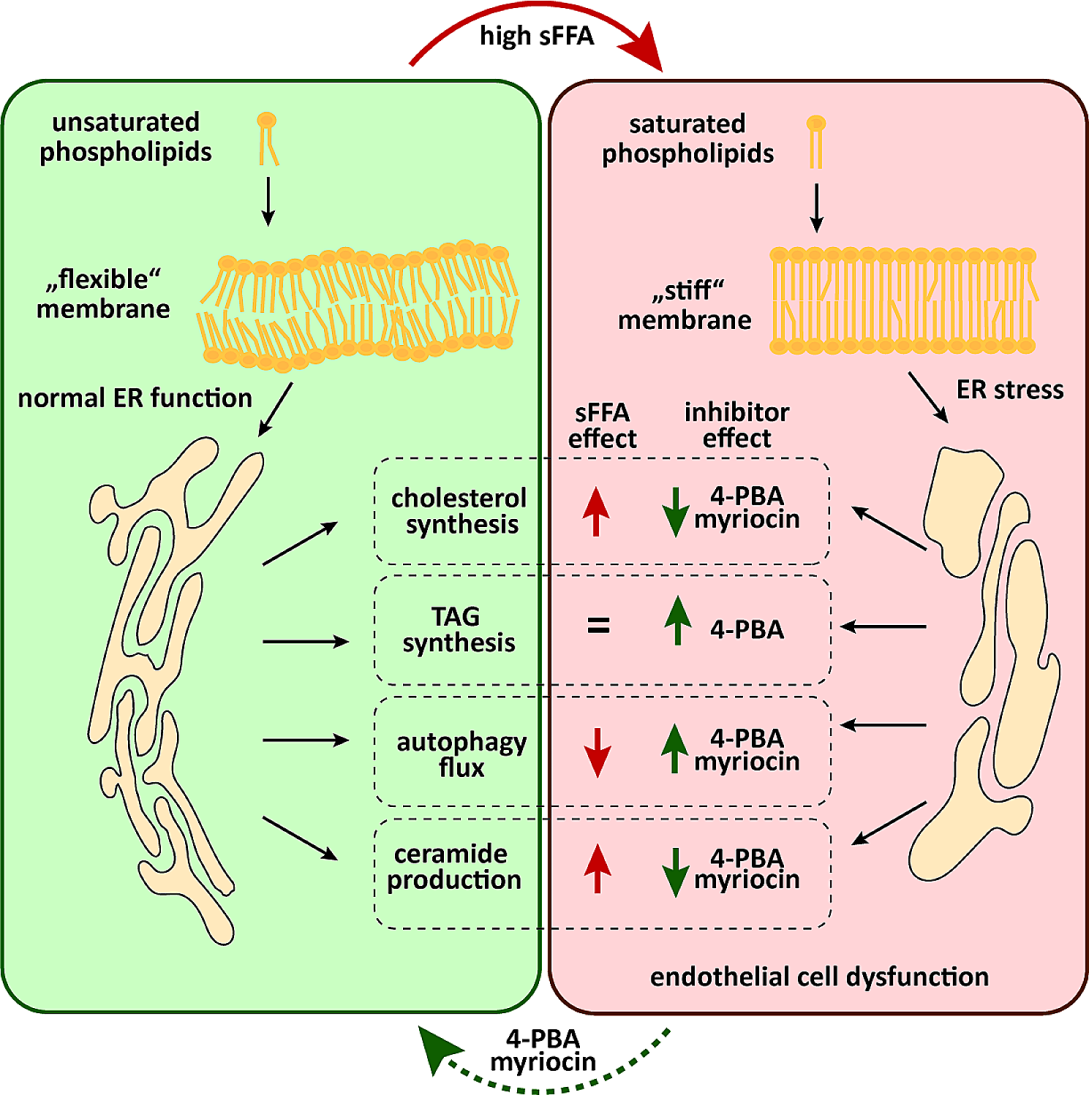
Graphical Summary sFFA disrupt the phospholipid composition of cellular membranes and change the membrane topology. These changes impair the ER function and thereby the lipid metabolism (e.g. increased cholesterol levels and enhanced ceramide synthesis) and autophagy flux in endothelial cells. Application of pharmacological ER-stress and ceramide synthesis inhibitors can restore negative effects of sFFA and improve endothelial cell function

We also investigated the synthesis of neutral lipids using lipidomics and results obtained from this analysis confirmed our findings on 4-PBA effects on TG synthesis and lipid droplet formation in previous experiments. Lipidomic analyis showed that beneficial effects of 4-PBA on palmitate-dependant lipotoxicity might rely on its potential to re-direct palmitate metabolism towards TG species which are consequently stored in lipid droplets. In agreement with this hypothesis, the levels of lipid droplet associated protein PLIN-2 were indeed increased upon treatment with 4-PBA alone. PLIN-2 protein levels are known to be induced upon synthesis of TGs and initial formation of lipid droplets [43], mostly due to impaired degradation and stabilization of the PLIN-2 protein [44]. Accordingly, our lipidomic analysis showed that the 4-PBA treatment alone or in combination with palmitate strongly enhanced TG synthesis and we propose that this effect of 4-PBA leads to increased expression of PLIN-2. In contrast, palmitate supplementation alone did not increase TG synthesis significantly in endothelial cells similar to results obtained in previous studies in hepatocytes [42]. Interestingly, 4-PBA strongly enhanced palmitate-induced synthesis of TGs with higly saturated acyl chains, whereas treatment with 4-PBA alone enhanced production of TGs with unsaturated acyl chains. Furthermore, we found that palmitate also strongly induced DG levels. Thus, both TG precursors, PtdOH and DG, were very significantly upregulated upon application of palmitate and 4-PBA addition effectively reduced these effects. Similar to our findings, enhanced synthesis of PtdOH and DG by palmitate was previously reported in hepatocytes and human muscles [42, 45]. These results suggest that palmitate causes stalling in TAG synthesis, possibly due to ER stress and UPR response. This stalling effect on TG synthesis pathway can be released upon co-application of 4-PBA resulting in restored TG synthesis and decreased incorporation of palmitate in phospholipids. Thus the effects of the 4-PBA on endothelial cell function most likely do not rely solely on clasiccal ER-stress inhibition, but might involve other effects of 4-PBA, such as induction of TG synthesis. In contrast, to effects of 4-PBA on TG production, inhibition of ceramide synthesis using myriocin did not show any effects on synthesis of TG, their precursors or phospholipid synthesis. Lipidomic analysis revealed that ceramides were around 50% increased in palmitate-treated cells and myriocin treatment decreased ceramide levels in both control condition and upon treatment with palmitate. Furthermore, we did not observe major effects of palmitate on sphingomyelin or glucosyl-/galactosylceramide levels. Closer inspection of specific ceramide species showed that ceramides 34:1;2, 36:1;2, 40:1;2 and 42:1;2 were all increased upon palmitate treatment and decreased when myriocin was co-applied. Serum abundance of these ceramide species carrying saturated fatty acids was correlated with the occurence of the major adverse cardiovascular event, cardiovascular mortality or insuline resistance recent studies [46–48]. Interestingly, 4-PBA also restored palmitate-dependent increase of total ceramides to control levels, demonstrating the central role of the ER function in palmitate-induced impairment in ceramide synthesis and general lipid metabolism. In conclusion, Fig. 9 summarizes our findings on effects of palmitate on lipid metabolism in endothelial cells. Our results suggest that the excess of sFFA leads to increased saturation in membrane phospholipids, which in turn inevitably disrupts the membrane fluidity and induces the ER stress-dependent UPR in endothelial cells. Whereas, general phospholipid saturation was not restored upon co-application of 4-PBA with palmitate, PtdOH saturation and all other adverse effect of palmitate like UPR induction, autophagy flux blockade, enhanced cholesterol and ceramide synthesis were partially reverted. Another important effect of 4-PBA was enhanced TG synthesis in palmitate-treated cells, reminiscent of previously described impact of oleate supplementation on re-direction of palmitate metabolism from PtdOH synthesis towards incorporation into TG in hepatocytes [42]. Our results once again stress the importance of the ER, as a central lipid metabolim hub, for the response of endothelial cells to palmitate. Furthermore, our work indicates that ceramide production due to impaired storage of excess palmitate, is a crucial step in sFFA-dependent disruption of endothelial cell function. Our data were generated in primary cell culture models and limited numbers of human serum samples, nevertheless our comprehensive analysis of palmitate-induced lipid metabolism in endothelial cells contributes to clarification of molecular mechanisms beyond sFFA and ceramide effects on cardiovascular system and its pathologies.

### Electronic supplementary material

Below is the link to the electronic supplementary material.

Supplementary Material 1

## Data Availability

No datasets were generated or analysed during the current study.
