# Supplementary material for: ER stress inhibition enhances formation of triacylglcerols and protects endothelial cells from lipotoxicity
Source: Cell Commun Signal. 2024 Jun 3;22:304. doi: 10.1186/s12964-024-01682-y (PMC11145897; doi:10.1186/s12964-024-01682-y)
Supplement: Supplementary file 1 — Supplementary Material 1 [file 12964_2024_1682_MOESM1_ESM.docx]

**Supplementary information**

**ER stress inhibition enhances formation of triacylglcerols and protects endothelial cells from lipotoxicity**

Igor Kovacevic^1,*^, Paula Schmidt^1^, Annkatrin Kowalski^1^, Bernd J. Helms^2^, Chris H.A. van de Lest^2^, Alexander Kluttig^3^, Guido Posern^1^

^1^Institute of Physiological Chemistry, Martin Luther University Halle-Wittenberg, 06128 Halle (Saale), Germany

^2^ Department Biomolecular Health Sciences, Veterinary Medicine, Utrecht University, 3584CM Utrecht, The Netherlands

^3^Institute of Medical Epidemiology, Biostatistics, and Informatics, Interdisciplinary Center for Health Sciences, Medical Faculty of the Martin Luther University Halle-Wittenberg, Halle (Saale), Germany.

* corresponding author

**Table S 1**

**Supplementary Table S 1.** Characteristics of the normal weight and obese study participants

| **Variable** | **Normal weight** (n=20) | | **Obese** (n=20) | ***P* value** |
| --- | --- | --- | --- | --- |
| cholesterol (mmol/l) | | 5.55 ± 0.77 | 5.56 ± 0.85 | 0.9640 |
| HDL (mmol/l) | | 1.49 ± 0.54 | 1.08 ± 0.24 | **0.0047** |
| LDL (mmol/l) | | 3.50 ± 0.70 | 3.58 ± 0.75 | 0.7221 |
| TG (mmol/l) | | 1.68 ± 1.10 | 2.23 ± 1.66 | 0.2336 |
| waist to hip ratio | | 0.90 ± 0.08 | 1.00 ± 0.09 | **0.0003** |
| HbA1c (%) | | 5.50 ± 0.28 | 5.62 ± 0.49 | 0.3199 |
| Glucose (mmol/l) | | 5.43 ± 0.58 | 5.84 ± 1.32 | 0.2195 |
| SBP (mmHg) | | 141.8 ± 21.36 | 141.65 ± 19.72 | 0.9822 |
| DBP 8mmHg) | | 85.43 ± 12.69 | 82.65 ± 11.03 | 0.4764 |
|  | |  |  |  |

Values in the table represent mean ± standard deviation. *P* values were calculated using unpaired t-test and *P* values under 0.05 are marked in bold. All laboratory parameters were measured in non-fasting venous blood samples as described in Greiser et al., 2009.

**Figure S1**

**
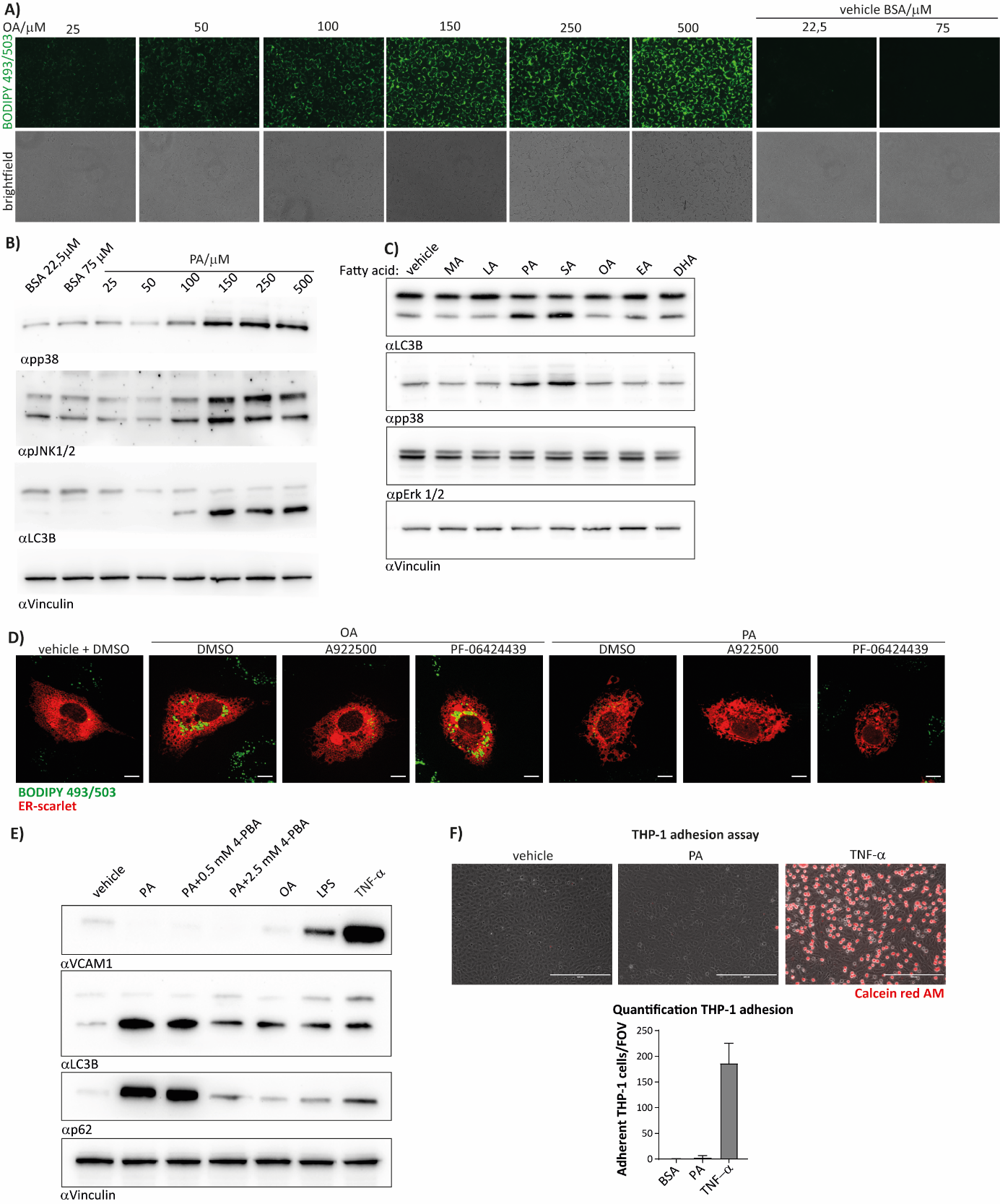
**

**Figure S1. a)** Oleate (OA) concentration-dependent induction of lipid droplet formation. HUVEC were treated with indicated OA concentrations or BSA as vehicle control for 16 hr. After the treatment the cells were stained using BODIPY 493/503 and imaged using EVOS® FL Cell Imaging System. **b)** Palmitate (PA) concentration-dependent signaling in endothelial cells. HUVEC were treated with indicated PA concentrations or BSA as vehicle control for 16 hr. Cells were lysed in 2x sample buffer and analysed for expression of LC3B, pp38 and pJNK1/2. Vinculin was used as loading control. **c)** Testing of signaling capacities of different fatty acid types in HUVEC. The cells were treated for 16 hr with 150 µM solutions of following fatty acids: lauric acid (LA), myristic acid (MA), palmitic acid (PA), stearic acid (SA), oleic acid (OA), elaidic acid (EA) or docosahexaenoic acid (DHA). Cells were lysed in 2x sample buffer and analysed for expression of LC3B, pp38 and pErk1/2. Vinculin was used as loading control. **d)** Influence of PA and OA on the ER morphology in endothelial cells. HUVEC were transfected with the Scarlet-ER fluorescent marker and treated with 150 µM PA or OA in combination with 5 µM A922500 or 10 µM PF-06424439. After the treatment cells were fixed, stained with BODIPY 493/503 and imaged using Zeiss AxioObserver fluorescence microsope. Scale bars represent 10 µm. e) Immunoblot analysis of VCAM-1 expression in HUVEC. The cells were treated with 150 µM PA alone or in combination with 0.5 mM or 2.5 mM 4-PBA, 150 µM OA, 1 µg/ml lipopolysaccharide (LPS) (Sigma) or 10 ng/ml TNF-α (Peprotech) for 16 hr. Cells were lysed in 2x sample buffer and analysed for expression of VCAM-1, LC3B and p62. Vinculin was used as loading control. f) THP-1 adhesion assay. Calcein Red AM labelled THP-1 cells were allowed to adhere on HUVEC pretreated with BSA, PA or 10 ng/ml TNF-α for 30 min. Non-adherent THP-1 cells were washed away and Calcein Red Am labelled adherent THP-1 cells were imaged using Evos FL microscope and 10x objective. Scale bars represent 400 um. Bar graph represents cell numbers calculated from 10 images per condition and error bars represent standard deviation (SD).

**Figure S2**

**
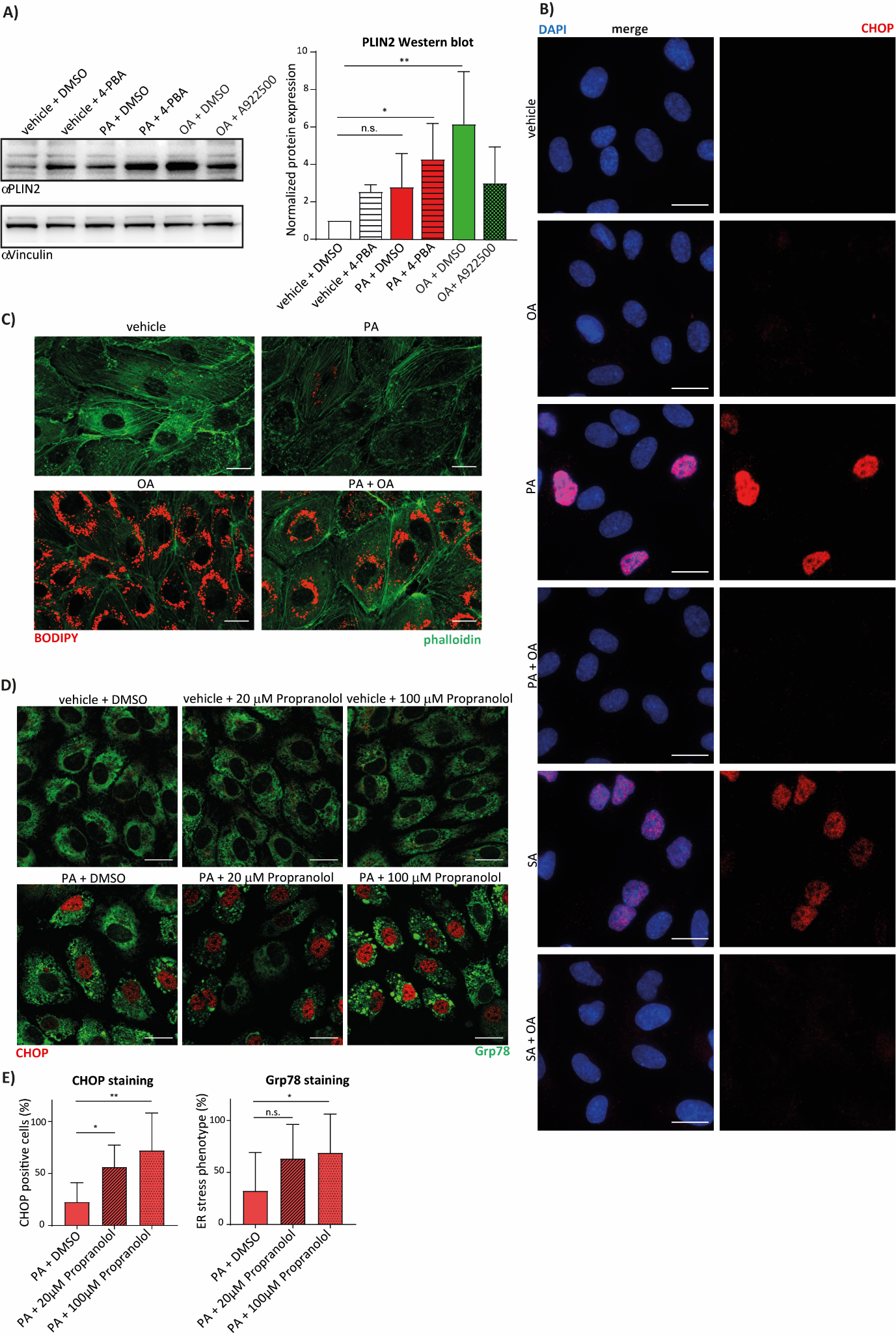
**

**Figure S2. a)** Immunoblot analysis of PLIN2 expression. HUVEC were treated for 16 hr with 150 µM PA alone or in combination with 2.5 mM 4-PBA, or with 150 µM OA alone or in combination with 5 µM A922500. Cells were lysed in 2X sample buffer and analysed for expression of PLIN2. Vinculin was used as loading control. Right side shows quantification of PLIN2 immunoblot. n=3. GraphPad Prism software and one-way ANOVA with Dunnett’s multiple comparison test were used for statistical analysis. b) Immunofluorescence analysis of CHOP expression in HUVEC treated for 16 hr with 150 µM PA or SA alone or in combination with 150 µM OA. Upon treatment, the cells were fixed and stained with CHOP antibody and DAPI and imaged using Zeiss AxioObserver fluorescence microsope. Scale bars represent 20 µm. **c)** BODIPY 493/503 staining of lipid droplets. HUVEC were seeded on 8-well IBIDI chamber and treated for 16 hr with 150 µM of PA, OA or combination of both. BSA alone was used as vehicle control. The cells were fixed, stained with BODIPY 493/503 and Phalloidin. The samples were imaged using Zeiss AxioObserver. Scale bar represents 20µm. **d)** Immunofluorescence analysis of CHOP expression and ER morphology in HUVEC treated for 16 hr with 150 µM PA alone or in combination with 20 µM or 100 µM propranolol. Upon treatment the cells were fixed and stained with CHOP and Grp78 antibodies and imaged using Zeiss AxioObserver fluorescence microsope. Scale bars represent 10 µm. **e)** Quantification of percentage of CHOP positive cells (n=3) and ER stress phenotype (n=4) in cells treated as in (c). Disrupted ER morphology with intensive punctuated staining of Grp78 was considered as manifestation of the ER stress phenotype. n=3. GraphPad Prism software and one-way ANOVA with Dunnett’s multiple comparison test were used for statistical analysis.

**Figure S3**

**
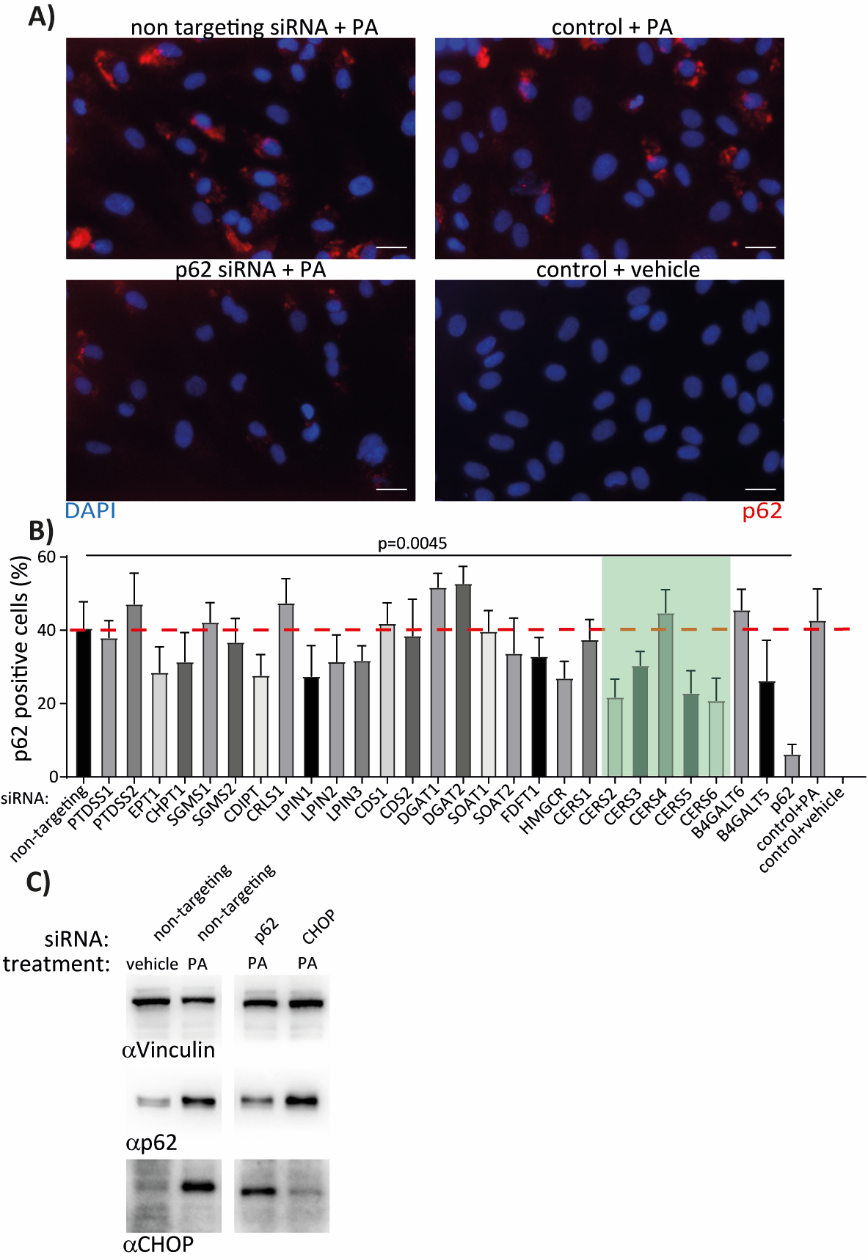
**

**Supplementary Figure 3. a)** Immunofluorescence analysis of p62 knockdown efficiency in endothelial cells. HUVEC were transfected with the control nontargeting siRNA pool, p62 siRNA pool or left untransfected. 48 hr post-transfection the cells were treated for 16 hr with 150 µM PA, fixed and stained with p62 antibody and DAPI. Scale bar represents 25 µm. **b)** Quantification of p62 positive cells in small-scale targeted siRNA screen in HUVEC. Cells were transfected with siRNA pools targeting genes indicated under the graph and treated as in (a). 3 images were analyzed per condition in three independent experiments. GraphPad Prism software and one-way ANOVA with Tukey’s multiple comparison test were used for statistical analysis. Error bars represent standard deviation (SD). Red dotted line represents the threshold percentage of p62 positive cells in PA treated non-targeting control cells. Green rectangle indicate the group of ceramide synthases with strongest decrease in number of p62 positive cells. **c)** Immunoblot control of p62 and CHOP expression in HUVEC transfected with nontargeting siRNA pool, or CHOP or p62 targeting siRNA pools and treated with 150 µM PA for 16 hr. Cells were lysed in 2X sample buffer and analyzed for expression of p62 and CHOP. Vinculin was used as loading control.
